# Adherence to Oral Antidiabetic Drugs in Patients with Type 2 Diabetes: Systematic Review and Meta-Analysis

**DOI:** 10.3390/jcm12051981

**Published:** 2023-03-02

**Authors:** Eugenia Piragine, Davide Petri, Alma Martelli, Vincenzo Calderone, Ersilia Lucenteforte

**Affiliations:** 1 Department of Pharmacy, University of Pisa, 56126 Pisa, Italy; 2 Specialization School in Hospital Pharmacy, University of Pisa, 56126 Pisa, Italy; 3 Department of Translational Research and New Technologies in Medicine and Surgery, University of Pisa, 56123 Pisa, Italy; 4 Department of Clinical and Experimental Medicine, University of Pisa, 56126 Pisa, Italy

**Keywords:** type 2 diabetes, T2D, therapeutic adherence, compliance, oral antidiabetic drugs, systematic review, meta-analysis

## Abstract

Poor adherence to oral antidiabetic drugs (OADs) in patients with type 2 diabetes (T2D) can lead to therapy failure and risk of complications. The aim of this study was to produce an adherence proportion to OADs and estimate the association between good adherence and good glycemic control in patients with T2D. We searched in MEDLINE, Scopus, and CENTRAL databases to find observational studies on therapeutic adherence in OAD users. We calculated the proportion of adherent patients to the total number of participants for each study and pooled study-specific adherence proportions using random effect models with Freeman–Tukey transformation. We also calculated the odds ratio (OR) of having good glycemic control and good adherence and pooled study-specific OR with the generic inverse variance method. A total of 156 studies (10,041,928 patients) were included in the systematic review and meta-analysis. The pooled proportion of adherent patients was 54% (95% confidence interval, CI: 51–58%). We observed a significant association between good glycemic control and good adherence (OR: 1.33; 95% CI: 1.17–1.51). This study demonstrated that adherence to OADs in patients with T2D is sub-optimal. Improving therapeutic adherence through health-promoting programs and prescription of personalized therapies could be an effective strategy to reduce the risk of complications.

## 1. Introduction

Type 2 diabetes (T2D) is one of the most common age-related metabolic disorders, affecting about 540 million people worldwide. It is associated with high morbidity and mortality, contributing to 6.7 million deaths in 2021 [1]. Recent projections indicate that global prevalence of T2D will increase exponentially in the next few years [2,3], pointing out the need for novel strategies to prevent the onset and progression of this disease. Indeed, chronic hyperglycemia enhances the risk of vascular inflammation, serious micro- and macrovascular alterations, and multiorgan dysfunction that leads to a large variety of diabetes complications (i.e., cardiovascular disorders, retinopathy, neuropathy, and nephropathy) [4,5]. These features make T2D a “multi-field” pathology, with a strong impact on public health and healthcare systems. In this regard, total costs for T2D management are projected to double in the next few years [6]. The increase in global prevalence and, in parallel, economic burden of T2D are impressive, and few definitions of T2D give a real idea of the “diabetes threat”. In recent times, T2D has been appropriately described as a “global preventable pandemic” [1], as it is a widespread disorder that can be partially prevented through lifestyle modifications [1,7]. Indeed, smoking, overweight/obesity, consumption of processed foods, and sedentary lifestyle are well-recognized exogenous risk factors for the onset of insulin resistance and, subsequently, T2D [8,9,10]. On the contrary, diet and exercise are cost-effective measures to prevent T2D and reduce overall mortality risk in patients with T2D [1]. 

Besides lifestyle changes, an effective strategy to reduce blood glucose levels is represented by antidiabetic drugs, which largely differ in their mechanism of action. According to the American Diabetes Association (ADA) guidelines [11], the biguanide metformin (MET), an AMPK (adenosine 5′-monophosphate-activated protein kinase) activator [12], is the first-line therapy for T2D. Furthermore, the use of MET in high-risk patients is emerging as a preventive strategy to reduce the incidence of T2D [13,14]. When monotherapy with MET does not lead to a significant reduction in glycated hemoglobin (HbA1c) levels, a combination therapy with other oral antidiabetic drugs (OADs) is recommended. If cost is a major issue, ADA guidelines indicate sulfonylureas (SULFs), glinides, and thiazolidinediones (TZDs) as second-line therapies [11]. SULFs and glinides are insulin secretagogues [15], while TZDs are the only OADs specifically treating insulin resistance by activating the peroxisome proliferator-activated receptor γ (PPAR-γ), but their use in clinical practice is limited due to skepticism about safety and tolerability [16,17]. α-glucosidase inhibitors (in monotherapy or in association with MET or SULFs) reduce intestinal absorption of dietary glucose, representing cost-effective strategies to improve glycemic control in newly diagnosed T2D patients [18]. Other commonly prescribed second-line therapies are glucagon-like peptide-1 receptor agonists (GLP-1RA) or the novel inhibitors of the renal sodium-glucose co-transporter-2 (SGLT-2i) [11]. SGLT-2i are insulin-independent OADs that can be used in patients with T2D and heart failure or in people experiencing gastrointestinal adverse effects due to MET. Indeed, SGLT-2i exhibits cardiovascular and renal effects beyond glycemic control that suggest further therapeutic uses for these drugs [19]. Finally, inhibitors of the dipeptidyl peptidase-4 enzyme (DPP-4i) are second-line drugs recently introduced in the pharmacotherapy of T2D [11]. DPP-4i preserve endogenous incretin hormones, such as GLP-1 (glucagon-like peptide-1) and GIP (gastric inhibitory peptide), from enzymatic hydrolysis, thus reducing blood glucose levels with a good tolerability profile [20]. However, when OADs fail to counteract serious β-cell dysfunction and/or insulin resistance, the add-on therapy with insulin or the replacement of OADs with insulin is necessary [11,21].

Despite the large number of OADs, adequate glycemic control (HbA1c < 7.0%) in patients with T2D is difficult to reach. A major cause of therapy failure is poor adherence (often referred to as “compliance”) to OADs, which plays a crucial role in the progression of T2D and risk of diabetes complications. Indeed, poor medication adherence is one of the most common causes of emergency room visits, hospitalization, enhanced morbidity and mortality, and increased costs of care in patients with T2D [22]. The current definition of adherence (i.e., the degree to which a patient correctly follows medical advice) does not fully describe the dynamic relationship between patient and physician. While the patient is often reluctant to engage in chronic antidiabetic therapy, government prevention programs and adherence supporting programs are not effective in enhancing medication adherence [1]. Presently, data about adherence to OADs are conflicting, and an updated summary of the literature is missing. Moreover, previous meta-analyses did not include the newest glucose-lowering drugs (i.e., DPP-4i and SGLT-2i). The aims of this systematic review and meta-analysis were to produce an updated adherence proportion to OADs in patients with T2D, assess the role of various factors (i.e., age, therapeutic regimen complexity, and therapeutic class) in determining global adherence, and investigate the association between good adherence and good glycemic control.

## 2. Materials and Methods

The protocol for this systematic review and meta-analysis has been registered in the PROSPERO database (CRD42021293269). The systematic review has been conducted following the PRISMA guidelines [23].

### 2.1. Inclusion Criteria

We included studies on patients with T2D, regardless of age, gender, and presence of co-morbidities. Exposure was the use of OADs, including biguanides, insulin secretagogues (i.e., sulfonylureas and glinides), thiazolidinediones (TZDs), α-glucosidase inhibitors, sodium-glucose co-transporter-2 inhibitors (SGLT-2i), and dipeptidyl peptidase-4 inhibitors (DPP-4i). The outcome was the measurement of therapeutic adherence as the number of adherent patients out of the total. We decided to focus on proportions of adherent patients to better describe the current problem of non-adherence, as previous meta-analyses on the same topic summarized data expressed as a percentage of medications taken or days covered by therapy without giving an immediate, easily interpretable picture of this real-world medical issue [24]. English-language observational studies (cohort and cross-sectional), published in the period 2011–2022, have been included.

### 2.2. Exclusion Criteria

We excluded studies that did not distinguish between injectable antidiabetic drugs and OADs, articles reporting outcomes other than adherence (i.e., persistence), and studies that did not measure adherence (or non-adherence) as number/proportion of adherent (or non-adherent) patients out of the total. We also excluded non-English-language studies, non-original (previously published) articles, abstracts, conference papers, short communications, reviews, and clinical trials.

### 2.3. Information Sources and Search Strategy

We searched MEDLINE (via Pubmed), SCOPUS, and CENTRAL (via the Cochrane library) for studies published from 2011 to 9 June 2022. The search strategy (Supplementary Material S1) was composed of three main terms. The first term was T2D, the second term was adherence or compliance, while the third term was OADs. The three terms were combined using the Boolean operator “AND”.

### 2.4. Selection Process

Two authors (EP and DP) independently screened titles and abstracts of studies identified by search strategies. Any discrepancies were discussed with a third reviewer, EL. Then, potentially relevant full texts were retrieved or, when not available, requested from the authors. Two authors (EP and DP) selected eligible studies, according to the inclusion and exclusion criteria, using the bibliographic management software Mendeley Desktop (version 1.19.6).

### 2.5. Data Extraction Process

The following information has been extracted: study design, data collection, aim of the study, and continent where the study was conducted; number and general characteristics of participants included age, gender, and definition of incident patients for studies reporting adherence in new users of OADs; period of inclusion and follow-up period; definition of adherent patients as reported in the study; number of adherent patients out of the total; reasons for non-adherence. Moreover, any data stratification (for age, gender, follow-up period for incident users, complexity of therapeutic regimen, and therapeutic class used) was retrieved. When available, the number of adherent (or non-adherent) patients with good glycemic control (HbA1c < 7.0%) or poor glycemic control (HbA1c ≥ 7.0%) has been extracted. The data extraction process was carried out by two authors (EP and DP) independently. Any disagreement was resolved with a third author, EL. Data were collected with the spreadsheet software Microsoft Excel (version 2102 build 13801.20864).

### 2.6. Risk of Bias (RoB) Assessment

Two authors (EP and DP) independently assessed the methodological quality of the included studies. Any disagreement was resolved with a third author, EL. The RoB of the included studies was evaluated with a modified version of the tool for prevalence studies developed by Hoy et al. [25], as previously reported [26]. The tool is composed of domains that consider the description of patient population, data collection method, total number of patients, and validity and reliability of the adherence calculation method employed. For each domain, a score of 0 (high risk of bias) or 1 (low risk of bias) was attributed. In total, domains were 9 for cross-sectional studies and 8 for cohort studies. Studies were classified at low risk of bias (total score: 8–9/9 or 7–8/8, respectively), moderate risk of bias (total score: 6–7/9 or 5–6/8, respectively), or high risk of bias (total score: 0–5/9 or 0–4/8, respectively). 

### 2.7. Effect Measures

We assessed the study-specific proportions of adherence to OADs by calculating the proportion of adherent patients to the total number of participants for each study. Appropriate calculations were performed when the study provided non-adherence or percentage of adherent patients. The association between good adherence and good glycemic control has been calculated as the odds ratio (OR). Briefly, the OR has been calculated as the ratio of two sets of odds: the odds of having good glycemic control among adherent patients divided by the odds of having good glycemic control among non-adherent patients.

### 2.8. Synthesis Methods

Data have been analyzed with the “metabin” and “metaprop” routines within the META and meta.DSL routine in RMETA package in R (version 4.12) [27]. A random effect model and the generic inverse variance method have been used to pool study-specific proportions of adherence. A random effect model with Freeman–Tukey transformation has been used to pool study-specific adherence proportions. Odds ratios (ORs) were meta-analyzed with a random effect model (DerSimonian and Laird method).

To quantify the heterogeneity, the Higgins heterogeneity index (I^2^) has been used. This index was categorized according to the Cochrane recommendations [28]. The heterogeneity was tested through Cochran’s Q test.

Subgroup analyses were performed according to adherence calculation method (use of questionnaires/verbal interviews or administrative data/pill counting), patient age and gender, follow-up period for incident users, complexity of therapeutic regimen, and therapeutic class used. Heterogeneity between groups and within groups was considered statistically significant if *p*-value < 0.10.

## 3. Results

### 3.1. Systematic Review

**Figure 1:**
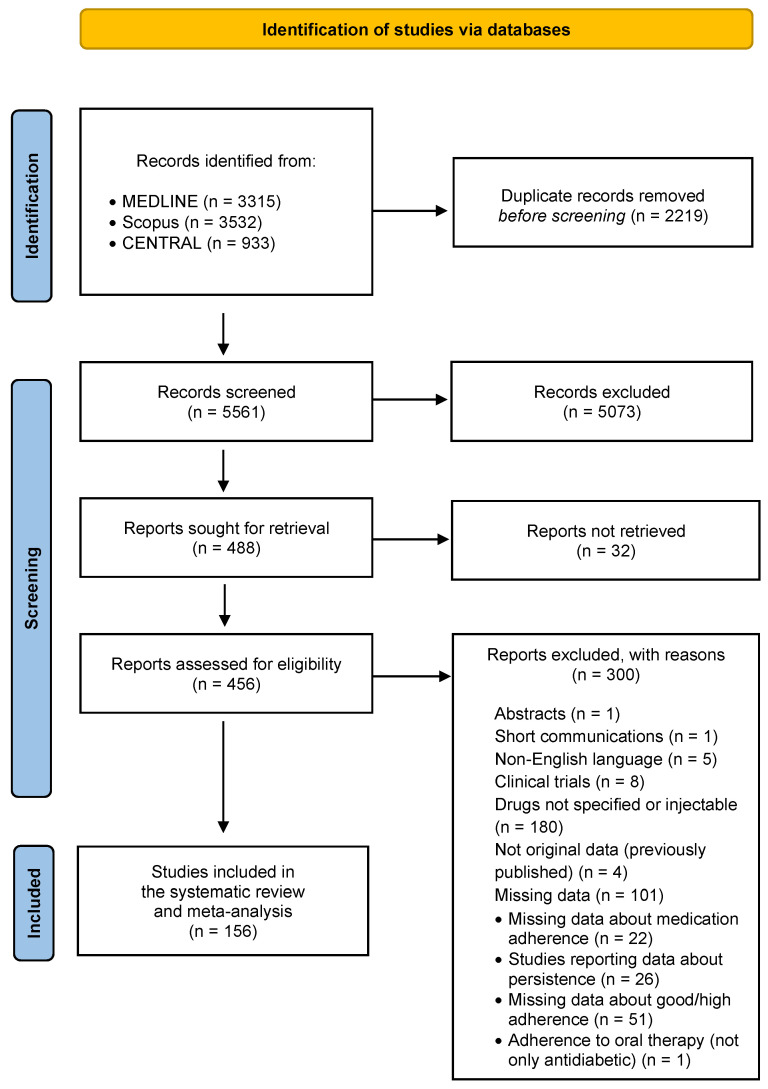
Flow chart of search.

Figure 1 represents the flow chart of search. We identified 3315 records through MEDLINE searching, 3532 records through Scopus searching, and 933 through CENTRAL searching. After the removal of duplicates, 5561 records were screened. Of them, 5073 were excluded based on information reported in titles and abstracts, and 488 were sought for retrieval. Then, the 456 available full texts were assessed for eligibility. In total, 156 studies (10,041,928 patients) met the inclusion criteria and were included in both qualitative and quantitative synthesis (meta-analysis) [29,30,31,32,33,34,35,36,37,38,39,40,41,42,43,44,45,46,47,48,49,50,51,52,53,54,55,56,57,58,59,60,61,62,63,64,65,66,67,68,69,70,71,72,73,74,75,76,77,78,79,80,81,82,83,84,85,86,87,88,89,90,91,92,93,94,95,96,97,98,99,100,101,102,103,104,105,106,107,108,109,110,111,112,113,114,115,116,117,118,119,120,121,122,123,124,125,126,127,128,129,130,131,132,133,134,135,136,137,138,139,140,141,142,143,144,145,146,147,148,149,150,151,152,153,154,155,156,157,158,159,160,161,162,163,164,165,166,167,168,169,170,171,172,173,174,175,176,177,178,179,180,181,182,183,184].

Among the included studies, 87 were cohort studies (10,013,130 patients) [42,43,44,47,48,49,50,51,52,53,54,55,56,57,59,62,65,66,67,68,69,70,71,72,73,74,77,79,83,84,85,87,89,90,93,95,96,97,101,105,106,107,108,110,112,113,114,118,119,120,121,122,123,124,125,126,128,129,130,133,137,141,142,143,144,148,149,150,151,153,154,156,157,158,159,161,163,164,165,167,168,172,174,175,178,179,181], while 69 were cross-sectional studies (28,798 patients) [29,30,31,32,33,34,35,36,37,38,39,40,41,45,46,58,60,61,63,64,75,76,78,80,81,82,86,88,91,92,94,98,99,100,102,103,104,109,111,115,116,117,127,131,132,134,135,136,138,139,140,145,146,147,152,155,160,162,166,169,170,171,173,176,177,180,182,183,184] (Table S1). The mean age of participants was 59 years, and about 50.5% of patients were men. A total of 41 studies presented data on incident users [43,48,49,50,51,52,54,55,56,62,65,66,67,70,71,74,83,84,85,97,101,104,105,106,107,114,118,119,123,124,125,126,128,129,133,150,153,154,161,165,167]. A total of 30 studies reported the number/proportion of adherent patients treated with biguanides [41,57,59,65,70,71,76,84,85,87,97,99,100,114,118,119,123,128,130,133,136,142,143,146,149,153,159,161,171,172], 23 with insulin secretagogues [41,43,52,59,65,66,70,71,76,84,97,99,123,128,133,136,142,146,149,159,165,171,172], 13 TZDs [41,59,65,66,70,71,84,123,133,142,143,149,159], 19 with DPP-4i [41,50,51,52,65,66,67,84,99,101,121,123,125,126,137,142,143,149,159], 8 with SGLT-2i [43,48,49,50,123,124,142,157], and 7 with α-glucosidase inhibitors [41,84,99,123,142,149,159]. All the studies (156) were included in the subgroup analysis by age and gender, 78 in the analysis by therapeutic regimen complexity [29,30,31,33,34,37,39,41,42,44,45,50,57,58,63,70,72,82,85,86,87,91,94,99,100,101,103,104,105,107,114,115,117,123,128,129,133,142,146,151,156,160,163,164,165,167,168,169,178,181,182], 47 in the analysis by follow-up period for incident users [43,48,49,50,51,52,54,55,56,65,66,67,71,74,83,84,85,97,101,104,105,106,107,114,119,123,124,125,126,128,129,133,150,154,161,165,167,173], and 16 studies were included in the analysis measuring the association between good adherence and blood glucose control [40,41,88,98,102,105,109,116,120,131,140,149,152,173,174,180]. As concerns the method of adherence measurement (Table 1), 65 studies used self-reported measurements (i.e., questionnaires and verbal interviews) [29,30,31,32,33,34,35,36,37,38,39,40,45,46,58,60,61,63,64,75,76,78,80,82,88,91,92,93,94,98,100,102,103,104,109,111,115,116,117,127,131,132,134,135,136,138,139,140,145,146,147,152,160,162,165,166,169,170,173,176,177,180,182,183,184], 3 studies used the pill-counting method [86,99,171], and 88 studies used administrative data [41,42,43,44,47,48,49,50,51,52,53,54,55,56,57,59,62,65,66,67,68,69,70,71,72,73,74,77,79,81,83,84,85,87,89,90,95,96,97,101,105,106,107,108,110,112,113,114,118,119,120,121,122,123,124,125,126,128,129,130,133,137,141,142,143,144,148,149,150,151,153,154,155,156,157,158,159,161,163,164,167,168,172,174,175,178,179,181]. 

A total of 34 studies reported the most common reasons for non-adherence to OADs, which were mainly forgetfulness, experiencing side effects, and high costs (Table 2).

### 3.2. Risk of Bias in Studies

Among cross-sectional studies (Table S2), 24 were considered at low risk of bias [29,35,36,39,40,63,76,82,88,98,102,103,109,111,115,116,136,138,145,147,166,180,182,184], 28 were endowed with moderate risk of bias [30,31,33,34,37,38,45,46,58,60,75,78,92,93,94,104,117,127,134,139,146,160,162,169,170,173,176,183], while 11 studies were at high risk of bias [32,64,80,91,100,131,132,135,140,152,177]. The high risk of bias was mainly due to the low number of patients, use of non-validated questionnaires, and specific characteristics of the included population that may have influenced adherence to OADs (i.e., inclusion of only young or old patients, only female or male patients, etc.). Among cohort studies (Table S3), 45 were classified as being at low risk of bias [41,43,44,49,50,56,57,65,66,67,68,69,73,81,85,87,89,90,96,99,110,113,118,121,122,124,125,126,128,129,130,133,137,142,149,151,154,157,158,161,163,164,172,174,178], 43 were considered at moderate risk of bias [42,48,51,52,53,55,59,62,70,71,72,74,77,79,83,84,95,97,101,105,106,107,108,112,114,119,120,123,143,144,148,150,153,155,156,159,165,167,168,171,175,179,181], while 5 studies were at high risk of bias [47,54,61,86,141]. In the latter studies, the main risk of bias was in the length of the period for measuring adherence, in the unacceptable definition of adherence, or in a target population that was not a close representation of the real-world population for gender, age, or presence of co-morbidities. 

### 3.3. Proportion of Adherent Patients to Therapy with OADs

The meta-analysis of 156 studies (10,041,928 patients) showed that the pooled proportion of adherent patients is 54% (95% confidence interval, CI: 51–58%), with considerable heterogeneity (I^2^ = 100.0%) (Figure S1). Therefore, we performed a sensitivity analysis according with risk of bias (Figure S2). The highest proportion of adherent patients was reported in studies at high risk of bias (63%; 95% CI: 48–77%; 499,464 patients), followed by studies at moderate risk of bias (57%; 95% CI: 52–61%; 2,484,177 patients) and low risk of bias (50%; 95% CI: 45–55%; 7,058,287 patients). The difference among subgroups was not significant (*p*-value = 0.10). 

### 3.4. Stratified Analyses

The analysis stratified by patient gender revealed that there was no significant difference between men and women in adherence to OADs (Figure S3). Proportions of adherent patients were 53% (95% CI: 46–60%) and 52% (95% CI: 44–59%), respectively. For age-stratified analysis, the patients were separated in three different categories according to tertiles of distribution. Proportions of adherent patients were 49% (95% CI: 43–55%) in people aged 35–56 years, 53% (95% CI: 47–59%) in people aged 56–63 years, and 58% (95% CI: 52–65%) in people aged 63–77 years. There was a certain tendency, without significance (*p*-value for heterogeneity among groups = 0.14), for having good adherence in older patients compared with younger patients (Figure S4). 

**Figure 2:**
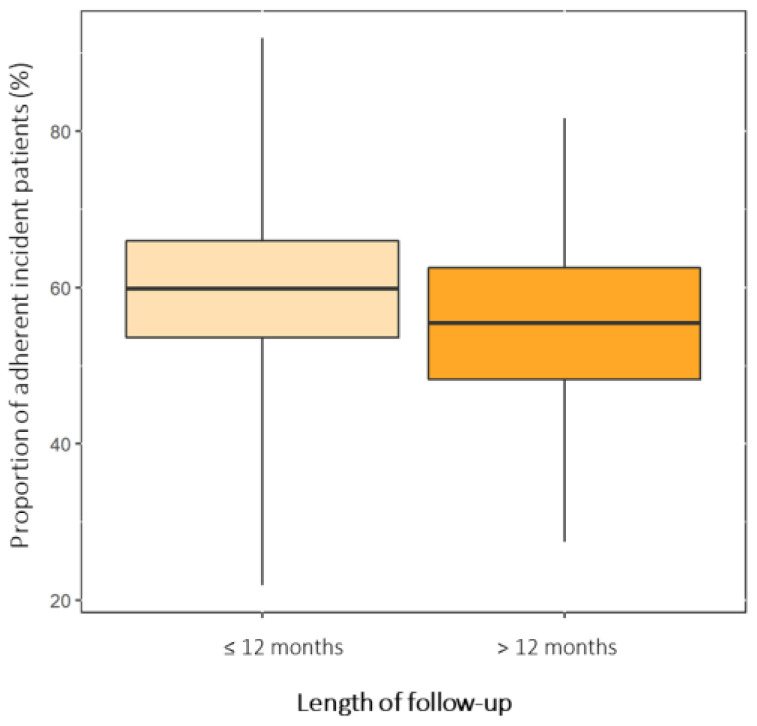
Pooled proportions of adherent incident patients stratified by length of follow-up. The width of boxes represents the 95% confidence intervals, while the whiskers extend for the 95% prediction intervals.

A total of 40 studies showed data about therapeutic adherence in new users of OADs (Figure 2 and Figure S5) [43,48,49,50,51,52,54,55,56,65,66,67,70,71,74,83,84,85,97,101,104,105,106,107,114,118,119,123,124,125,126,128,129,133,150,153,154,161,165,167]. These studies reported an overall adherence proportion of 58% (95% CI: 53–64%), which was measured in different follow-up periods. In particular, 34 study-arms calculated the number of adherent patients in a period less than or equal to 12 months and 11 study-arms in a period greater than 12 months (from 18 months to 7 years). The results of the stratified analysis by follow-up period showed there was no difference among subgroups (*p*-value = 0.43). The proportion of adherent patients was 59% (95% CI: 53–66%) in a follow-up period less than or equal to 12 months and 55% (95% CI: 48–63%) in a follow-up period greater than 12 months.

**Figure 3:**
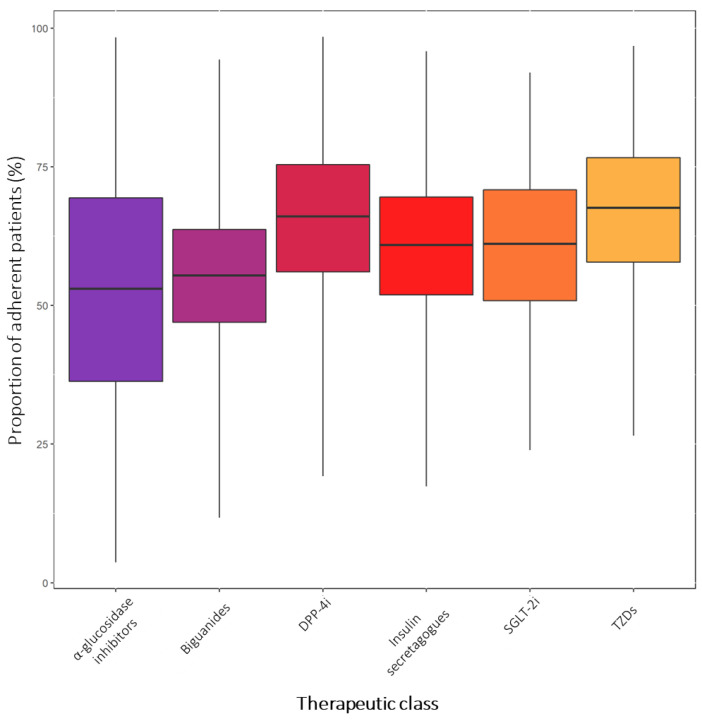
Boxplot representing the pooled proportions of adherent patients (expressed as a percentage) stratified by therapeutic class. The boxes represent the 95% confidence intervals of the proportions, while the whiskers extend for the 95% prediction interval.

As concerns the therapeutic class of OADs used, the difference among groups was not significant (*p*-value = 0.37) (Figure 3 and Figure S6). The highest adherence proportion has been found for TZDs (68%, 95% CI: 58–77%), followed by DPP-4i (66%, 95% CI: 56–75%), SGLT-2i (61%, 95% CI: 51–71%), insulin secretagogues (61%, 95% CI: 52–70%), biguanides (55%, 95% CI: 47–64%), and α-glucosidase inhibitors (53%, 95% CI: 36–69%). Most studies investigated adherence to biguanides (30 study-arms) [41,57,59,65,70,71,76,84,85,87,97,99,100,114,118,119,123,128,130,133,136,142,143,146,149,153,159,161,171,172], insulin secretagogues (23 study-arms) [41,43,52,59,65,66,70,71,76,84,97,99,123,128,133,136,142,146,149,159,165,171,172], and DPP-4i (19 study-arms) [41,50,51,52,65,66,67,84,99,101,121,123,125,126,137,142,143,149,159]. Conversely, few studies evaluated adherence to TZDs (13 study-arms) [41,59,65,66,70,71,84,123,133,142,143,149,159], SGLT-2i (8 study-arms) [43,48,49,50,123,124,142,157], and α-glucosidase inhibitors (7 study-arms) [41,84,99,123,142,149,159].

In the stratified analysis by complexity of the therapeutic regimen, we have not found a significant difference among patients in monotherapy or combination therapy (with or without insulin) (*p*-value for heterogeneity among groups = 0.28) (Figure S7).

Finally, we stratified the results by the method of adherence measurement, which ranged from questionnaires and verbal interviews (66 studies) to the use of administrative data and pill-counting methods (90 studies). This stratification was performed to evaluate whether data collection might have influenced the outcome of the study. We found that the proportion of adherent patients calculated by surveys was lower than that measured by means of administrative data (i.e., MPR and PDC) or pill counting (49%, CI: 43–55% vs. 58%, CI: 54–62%, respectively) (Figure S8). The difference among subgroups was significant (*p*-value = 0.01).

### 3.5. Association between Good Adherence and Blood Glucose Control

**Figure 4:**
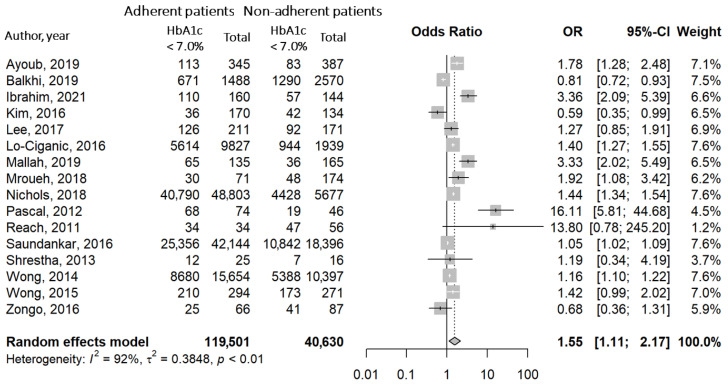
Forest plot with OR and relative confidence intervals for the association between good adherence and good glycemic control (HbA1c < 7.0%). White lines: confidence interval within the square. Black lines: confidence interval crossing the bounds of the square [40,41,88,98,102,105,109,116,120,131,140,149,152,174,173,180].

The meta-analysis evaluating the possible association between good adherence to OADs and good glycemic control (HbA1c < 7.0%) was performed on 16 studies [40,41,88,98,102,105,109,116,120,131,140,149,152,173,174,180]. Results showed an interesting scenario, that is, a positive and significant association between therapeutic adherence and levels of HbA1c < 7.0% (OR = 1.55, CI: 1.11–2.17) (Figure 4). This result, although preliminary, suggests that adherence to OADs is associated with glycemic control.

## 4. Discussion

Poor adherence to oral antidiabetic therapy represents a major barrier to successful management of T2D and strongly contributes to the impressive economic burden of diabetes. Indeed, the main consequence of sub-optimal adherence is the enhanced risk of diabetes complications, hospitalization for major disorders, and death [1,4,5], which impact on both health expenditure and efficiency of the healthcare systems [185]. In this scenario, an updated analysis of adherence proportions to OADs could guide clinicians in the development of new tools/strategies to improve therapeutic adherence in patients with T2D.

In this work, we systematically reviewed data from 156 studies including 10,041,928 patients with T2D. First, we reported that 54% (95% CI: 51–58%) of patients using OADs are adherent to the prescribed therapy. This result is perfectly in line with previous studies evaluating adherence to therapy in patients with other chronic pathological conditions, such as cardiovascular diseases [186,187].

The most commonly reported reasons for non-adherence are forgetfulness and experiencing side effects. In this regard, many patients intentionally decide not to adhere to their medication when they experience side effects, without informing the general practitioner. This behavior is alarming and stresses the need to strengthen the relationship between patients and healthcare providers to increase therapeutic adherence. A third barrier to adherence is the cost of treatment. Of note, the pricing and reimbursement policy of OADs largely differs among healthcare systems. The economic issue could influence patients’ compliance with oral antidiabetic therapy and could have contributed to the high heterogeneity observed in our analysis [188,189]. Furthermore, the absence of clinical signs and symptoms in the progression of T2D contributes to the low perception of both risk of complications and long-term consequences of non-compliance by patients with T2D. Therefore, the identification of new tools for the management of chronicity, aimed at improving both patient awareness of the disease and collaboration among healthcare providers (i.e., diabetologists, general practitioners, pharmacists, and nurses), is essential to promote therapeutic adherence [190,191]. For instance, many clinical studies demonstrated that the counselling activity of pharmacists improves adherence to chronic therapies and reduces costs for the healthcare systems [192,193]. Furthermore, the results of a recent systematic review with meta-analysis showed that pharmacy-led interventions increase adherence to antidiabetic therapy and enhance blood glucose control in patients with T2D [194]. This confirms the central role of pharmacists in monitoring therapeutic adherence. 

Given the wide heterogeneity observed in the global analysis, we performed subgroup analyses stratified by gender and age to investigate their potential role in determining poor therapeutic adherence. In previous studies, a certain tendency for having good therapeutic adherence has been associated with the male gender [110,195,196]. We showed that there is no significant difference in medication adherence between men and women. This was unexpected, as others have reported that women are less adherent than men to chronic therapies [110,195,196]. However, a common limitation of primary studies is the low number of patients, which does not allow the conclusive explanation of the role of gender in determining adherence to antidiabetic therapy. On the contrary, our work summarized data obtained from 10,041,928 patients and, even if with some limitations, it furnishes a more precise picture of the real-world population. 

Interestingly, there were slight differences in adherence to OADs between patients with different ages. Indeed, older patients appeared quite more adherent than younger patients (53% vs. 49%). This tendency, although not statistically significant, might derive from two crucial aspects. On one hand, aged patients are more aware of the chronicity of T2D and risk of complications, due to personal experiences, and they usually have family support [197]. On the other hand, clinicians generally prescribe to aged patients OADs with a lower risk of adverse effects (i.e., hypoglycemia) [21]. Evidence about therapeutic adherence stratified by age is quite conflicting and further studies are required to better elucidate this aspect. In any case, the poor adherence to OADs in young patients is alarming, as T2D is becoming a widespread disease in people aged less than 35–40 years. This mainly results from the large consumption of Western diets and the sedentary lifestyle, as well as from genetic and environmental factors, which lead to the premature onset of obesity and other metabolic disorders, including T2D [198]. 

We also investigated the proportions of adherent incident patients (new users) stratified by length of follow-up period (≤12 months and >12 months). The results of this subgroup analysis showed that there is no significant difference in the therapeutic adherence between the two periods of follow-up. After the first year of therapy, the proportion of adherent incident patients is almost superimposable to that of the general population (i.e., 55%). This tendency is expected as, in parallel with time, both impairment in quality of life and the incidence of adverse events increase. Importantly, new users of OADs are generally young adult patients with an active social and working life who might have a distorted idea of the risk–benefit profile of OADs [199]. Therefore, in this vulnerable group of patients, the personalization of therapy and the introduction of programs aimed at increasing adherence to OADs is essential to prevent early discontinuations and reduce the risk of diabetic complications from the earliest stages of this “devious disease”. In this regard, the use of dedicated smartphone apps may have high applicability in clinical practice among young adult people with T2D, but the usefulness of this tool is still unclear [200,201,202].

A recent meta-analysis showed that therapeutic adherence also differs between patients using different OADs [24]. In our analysis, a similar tendency has been reported, confirming the results by McGovern and colleagues [24]. α-glucosidase inhibitors showed the worst proportion of adherent patients (53%), followed by biguanides (55%), insulin secretagogues and SGLT-2i (both 61%), DPP-4i (66%), and TZDs (68%). A similar trend in adherence proportions has also been reported in the meta-analysis by McGovern et al. [24], who, however, did not investigate therapeutic adherence to the new antidiabetic drugs SGLT2-i. Therefore, our analysis confirms the previous results and adds something new in this dynamic and complex scenario. Such differences in adherence proportions could be attributed to the higher risk of adverse effects associated with the regular use of α-glucosidase inhibitors, biguanides, and insulin secretagogues compared with the most recent OADs. For instance, prolonged therapy with α-glucosidase inhibitors can lead to mild gastrointestinal symptoms (such as flatulence and diarrhea) [18]. Similarly, therapeutic use of biguanides is associated with increased risk of gastrointestinal disorders (i.e., nausea, vomiting, abdominal pain, and diarrhea) [203], which might account for the low adherence to biguanides. The most common side effects reported by users of insulin secretagogues, instead, are weight gain and hypoglycemic events, sometimes leading to hospitalization [204,205]. It is worth noting that insulin secretagogues are usually prescribed to young adult patients (i.e., patients with pre-diabetes or a new diagnosis of T2D) due to the enhanced risk of hypoglycemia in aged people. This could be a further reason of sub-optimal adherence to insulin secretagogues, as young patients usually lack awareness of the long-term consequences of non-compliance and intentionally decide to stop therapy when they experience side effects. On the contrary, SGLT2-i, TZDs, and DPP-4i are second- and third-line therapies usually prescribed in the advanced stages of T2D, when patients seem to have higher adherence to OADs. This is compatible with previous results on patients using long-term therapies, such as oral anticoagulants [206] and biological drugs [207]. Moreover, the newest antidiabetic drugs show good tolerability, as the incidence of adverse events associated with their regular use is quite low [208]. Among SGLT-2i users, the most commonly reported side effects are urinary tract infections and increased urination [209], while DPP-4i users can experience headache and hypersensitivity reactions without consistent risk of gastrointestinal disorders [210,211,212]. Finally, data on TZDs derive from a relatively low number of patients compared with users of other drugs (123, 301). This was expected, as TZDs are considered “forgotten” OADs, which are less frequently prescribed due to growing concerns about their safety profile. Indeed, prolonged use of TZDs seems to be associated with enhanced risk of fluid retention, heart failure, peripheral fractures, and weight gain [17]. The low number of TZD users represents a limitation of our analysis and does not allow us to directly compare the adherence proportion with that calculated for the other groups of OAD users, which are more numerous.

Our analysis suggests that adherence to oral antidiabetic therapy can also depend on the complexity of the therapeutic regimen. The proportion of adherent patients receiving polytherapy (i.e., combination of OADs or OADs plus insulin) is markedly reduced compared with patients receiving monotherapy (57% for monotherapy vs. 48% for combination therapy plus insulin). This could result from patients’ reluctance towards the use of multiple drugs or, in severe cases, injection therapies. However, the increasing use of combination pills in clinical practice introduces a potential bias in our analysis [213]. Indeed, most of the included studies did not specify the characteristics of the combination therapy (i.e., use of one or more pills), thus limiting conclusive comparisons between patients receiving different therapeutic regimens. 

Finally, we separately analyzed adherence proportions by method of adherence measurement. There are many indirect measurements of adherence, which include the use of research and administrative data (i.e., calculation of MPR and PDC values), as well as methods applied in patient care settings (i.e., self-reported questionnaires and verbal interviews) [214]. Results showed that the proportion of adherent patients obtained from administrative data and pill-counting methods was 58%, while the adherence proportion calculated with questionnaires and verbal interviews was 49%. This evidence indicates that the use of administrative data for calculating adherence to chronic therapy could lead to missing some crucial aspects. Indeed, it does not take in account changes in therapeutic regimens (i.e., dosages, personalization of therapy, etc.) deriving from medical decisions, as well as barriers associated with non-adherence. Conversely, the use of questionnaires and interviews might better reflect the patient’s attitude towards antidiabetic therapy, but results can be partially distorted due to both non-response and selection bias [215]. Finally, pill counting is a low-cost measurement that does not give specific information about patterns of adherence or proper use of the drug. Moreover, as for administrative data, this method does not consider potential changes in the dosing regimen [214].

Blood glucose control (HbA1c < 7.0%) is a timely challenge for public health systems. Indeed, chronic hyperglycemia leads to diabetes complications, hospitalization, and increased costs [4,5]. In this study, we reported a significant direct association between good adherence to OADs and good glycemic control (OR = 1.55). Although the direction is still not clear, this association strengthens the hypothesis that good adherence to therapy might reduce hyperglycemia in patients with T2D and slow down the progression of disease [216,217]. A similar association between therapeutic adherence and clinical goals has been previously observed for other chronic diseases, such as hypertension [218] and chronic kidney disease [219].

## 5. Conclusions

Adherence to OADs in patients with T2D is sub-optimal and must be improved. Increasing evidence suggests that poor adherence is associated with chronic hyperglycemia, as well as enhanced risk of diabetes complications and hospitalization [1]. Therefore, health education programs aimed at enhancing therapeutic adherence among patients with T2D could represent effective strategies to slow down the progression of disease and reduce healthcare costs. However, healthcare providers are often reluctant to focus on prevention, and the tools proposed so far have not been applied in clinical practice. 

In this study, we highlighted the complexity of the “therapeutic adherence challenge”, which is the result of both medical factors and psychosocial determinants. Therefore, the old rule of a “stringent therapeutic scheme for all patients” must be updated in light of current medical need. Moreover, the definition of individualized glycemic targets is fundamental to obtain valid clinical goals. Therefore, the choice of therapy from physicians must consider individual characteristics, personal preferences, life expectancy, presence of co-morbidities, and the risk/benefit profile of OADs. For instance, biguanides should not be prescribed to people at risk of lactic acidosis (such as patients with kidney failure) or folate deficiency, and they should be introduced at low doses to improve patients’ tolerance. Sulfonylureas, on the other hand, are contraindicated in patients with renal and hepatic diseases or at risk of hypoglycemia [220]. In general, adherence seems to be higher in patients using the newest drugs (i.e., DPP-4i and SGLT-2i) instead of the oldest OADs, as previously reported for other chronic therapies [221]. 

To the best of our knowledge, this is the first systematic review reporting the proportion of patients with “good adherence” rather than the percentage of medications taken in a defined period or the number of days covered by drug therapy [222], thus describing the medical problem of poor adherence from an alternative and more impactful perspective. Moreover, this is the first study exclusively focusing on OADs, partially overcoming the bias associated with the heterogeneous route of administration of antidiabetic drugs. Additional strengths are the numerosity of patient population (10,041,928 subjects), the high number of included studies (156), as well as the inclusion of the more recent OADs (i.e., SGLT-2i and DPP-4i). Stratified analysis by the adherence calculation method allowed us to reduce the heterogeneity of the global analysis, also leading to a better comprehension of both potentiality and limitations of the indirect methods for measuring medication adherence. The subgroup analyses (by gender, age, type of OAD, and complexity of the therapeutic regimen) provided an updated “picture” of determinants of good adherence in the general population, which is fundamental to promote personalized therapies and intervention programs aimed at improving adherence to antidiabetic therapy. Finally, the meta-analysis of association between good adherence and glycemic control further suggested the crucial role of patient compliance in the progression of T2D. 

Limitations of our analysis include the wide heterogeneity in adherence measurement periods, which ranged from a few weeks to 7 years, as well as in patient characteristics (i.e., duration of diabetes, presence of comorbidities, polytherapy, etc.). However, this heterogeneity fully reflects the real-world population, giving a real picture of the ongoing problem of poor therapeutic adherence in patients with T2D. The lack of information on the role of ethnicity in determining therapeutic adherence is another limitation of our study. Very few articles included in our systematic review reported adherence data stratified by race/ethnicity, thus not allowing us to perform a meta-analysis. This is a common limitation of studies using administrative data, as electronic databases do not capture relevant sociodemographic details that may influence therapeutic adherence, including race/ethnicity. Finally, we limited our search to the period 2011–2022, thus excluding all previously published studies. However, contextualizing our research in a relatively short period has allowed us to produce an adherence proportion that fully reflects the current behavior of patients with T2D towards OADs, leading to conclusions that can be generalized to the real-world population, avoiding the risk of misinterpretation of the results. 

## Figures and Tables

**Table 1:** Details of adherence calculation methods. List of abbreviations: MMAS-8: eight-item Morisky Medication Adherence Scale; MMAS-4: four-item Morisky Medication Adherence Scale; LMAS-14: Lebanese Medication Adherence Scale; MCQ: Medication Compliance Questionnaire Adherence; MARS: Medication Adherence Report Scale; ARMS: Adherence to Refills and Medications Scale; PDC: proportion of days covered; MPR: medication possession ratio. * Some studies reported more than one follow-up period.

Good/High Adherence Definition/Calculation	Number of Studies
Self-reported (65)	
MMAS-8 questionnaire Score = 8 [30,35,64,76,91,103,104,111,115,135,160,169,180] Score ≥ 6 [39,58,145,173,176,183] Score ≥ 7 [78] Score > 8 [30]	21 13 6 1 1
MMAS-4 questionnaire Score = 4 [63,75,94,138,146,184] Score > 3 [46]	7 6 1
LMAS-14 questionnaire Score ≥ 38 [40,116] Score > 11 [88]	3 2 1
MCQ questionnaire (score > 27) [29,34,37]	3
MARS questionnaire Score > 20 [60] Score = 25 [102]	2 1 1
ARMS questionnaire (score = 12) [38,98]	2
Verbal interview >80% of the prescribed antidiabetic medications taken [61,92,93,117] >90% of the prescribed antidiabetic medications taken [177] Compliance ≥ 90% [165]	6 4 1 1
Other questionnaires ^a^ [31,32,33,36,37,45,80,82,100,109,127,131,132,134,136,139,140,147,162,166,170]	21
Pill counting ^b^ (3) [86,99,171]	
Use of administrative data (88)	
PDC ≥ 80% [42,43,44,47,48,49,50,51,52,59,62,66,67,68,69,70,71,73,77,84,87,89,90,95,96,97,101,105,106,112,114,118,120,122,123,124,125,126,130,137,144,148,149,150,155,157,161,163,164,178,179] Follow-up period: * <12 months (1–9 months) =12 months >12 months (24–48 months) Not specified	51 5 35 13 4
MPR ≥ 80% [53,54,55,56,57,65,72,74,83,85,107,110,113,128,129,133,141,143,151,153,154,158,167,168,172,174] Follow-up period: * <12 months (3–6 months) =2 months >12 months (18 months–7 years) Not specified	26 4 15 9 3
Other calculations ^a^ [41,79,81,108,119,121,142,156,159,175,181]	11

^a^ More details can be found in Table S1. ^b^ 90–105% of the prescribed medication taken [86]; 90–100% of the prescribed medication taken [171]; prescription reduction ratio (PRR) < 0.2, calculated by measuring the number of drugs originally prescribed and the reduced amount after use of leftover drugs [99].

**Table 2:** Most reported reasons for non-adherence (barriers to adherence). ^1^: also includes forgetfulness due to events that disrupt the routine of taking medicine as prescribed (i.e., being away for long time, having someone at home, being at restaurant, travelling). ^2^: problems in following the treatment plan, ignorance of life-long drug adherence, non-awareness of the chronic nature of diabetes, dislike of the taste of the medication, difficulty in swallowing due to the size of medication.

Reasons for Non-Adherence	Number of Studies
Forgetfulness ^1^	28
Experiencing of side effects	15
High costs	12
Absence/disappearance of symptoms	9
Carelessness	8
Multiple medications	6
Procrastinating on refills or renewals of prescriptions	6
Feeling hassled or bored to take medication regularly	5
Worry about using medication regularly due to possible risk of unintended effects	5
Not accepting disease/feeling disease under control	4
Lack of confidence or poor communication with the physician/healthcare provider	4
Being too busy/time constraints	4
Social influence and environmental context	3
Complexity of treatment regimen	2
Inefficacy	2
Preference for herbal remedies	2
Others ^2^	5

## Data Availability

The data that support the findings of this study are available from the corresponding author upon reasonable request.

## Supplementary Materials

The following supporting information can be downloaded at: https://www.mdpi.com/article/10.3390/jcm12051981/s1, Supplementary Material S1: Search strategy, Table S1: Characteristics of studies included in the systematic review; Table S2: Risk of bias of cross-sectional studies, Table S3: Risk of bias of cohort studies; Figure S1: Forest plot of proportions of adherent patients to OADs and relative confidence intervals; Figure S2: Forest plot of proportions, and relative confidence intervals, according with risk of bias; Figure S3: Forest plot of proportions, and relative confidence intervals, of adherent patients stratified for gender; Figure S4: Forest plot of proportions, and relative confidence intervals, of adherent patients stratified for age category; Figure S5: Forest plot of proportions, and relative confidence intervals, of adherent incident patients stratified for follow-up period; Figure S6: Forest plot of proportions, and relative confidence intervals, of adherent patients stratified for therapeutic class; Figure S7: Forest plot of proportions, and relative confidence intervals, of adherent patients stratified for therapeutic regimen complexity, Figure S8: Forest plot of proportions, and relative confidence intervals, of adherent patients stratified for method of adherence measurement.
