# Supplementary material for: Adherence to Oral Antidiabetic Drugs in Patients with Type 2 Diabetes: Systematic Review and Meta-Analysis
Source: J Clin Med. 2023 Mar 2;12(5):1981. doi: 10.3390/jcm12051981 (PMC10004070; doi:10.3390/jcm12051981)

Supplementary Material S1. Search strategy

MEDLINE (via Pubmed)

((T2DM OR (type AND 2 AND diabetes) OR (diabetes AND mellitus) OR diabetes OR (type AND 2 AND diabetes AND mellitus)) AND (adherence OR compliance) AND ((antidiabetic AND (agent OR drug*)) OR (glucose AND lowering AND (agent* OR drug*)) OR (antihyperglycemic AND (agent* or drug*)) OR tolbutamide OR chlorpropamide OR glibenclamide OR glimepiride OR glipizide OR gliclazide OR gliquidone OR repaglinide OR nateglinide OR mitiglinide OR pioglitazone OR semaglutide OR vildagliptin OR sitagliptin OR saxagliptin OR linagliptin OR alogliptin OR metformin OR dapagliflozin OR canagliflozin OR empagliflozin OR sulphonylurea* OR thiazolidindione* OR glitazone* OR (GLP-1 AND receptor AND agonist*) OR (DPP-4 AND inhibitor*) OR gliptin* OR (SGLT2 AND inhibitor*))*

Scopus

(TITLE-ABS-KEY (t2dm) OR TITLE-ABS-KEY (type AND 2 AND diabetes) OR TITLE-ABS-KEY (diabetes AND mellitus) OR TITLE-ABS-KEY (diabetes) OR TITLE-ABS-KEY (type AND 2 AND diabetes AND mellitus) AND TITLE-ABS-KEY (adherence) OR TITLE-ABS-KEY (compliance) AND TITLE-ABS-KEY (antidiabetic AND agent OR drug*) OR TITLE-ABS-KEY (glucose AND lowering AND agent* OR drug*) OR TITLE-ABS-KEY (antihyperglycemic AND agent* OR drug*) OR TITLE-ABS-KEY (tolbutamide OR chlorpropamide OR glibenclamide OR glimepiride OR glipizide OR gliclazide OR gliquidone OR repaglinide OR nateglinide OR mitiglinide OR pioglitazone) OR TITLE-ABS-KEY (semaglutide OR vildagliptin OR sitagliptin OR saxagliptin OR linagliprin OR alogliptin OR metformin) OR TITLE-ABS-KEY (dapagliflozin OR canagliflozin OR empagliflozin OR sulphonylurea* OR thiazolidindione* OR glitazone*) OR TITLE-ABS-KEY (glp-1 AND receptor AND agonist*) OR TITLE-ABS-KEY (ddp-4 AND inhibitor*) OR TITLE-ABS-KEY (gliptin*) OR TITLE-ABS-KEY (sglt2 AND inhibitor*)) AND (LIMIT-TO (DOCTYPE , "ar")) AND (LIMIT-TO (LANGUAGE , "English"))*

CENTRAL (via the Cochrane Library)

ID	Search
#1	("T2DM"):ti,ab,kw
#2	(type):ti,ab,kw AND (2):ti,ab,kw AND ("diabetes"):ti,ab,kw
#3	("diabetes"):ti,ab,kw AND (mellitus):ti,ab,kw
#4	#1 OR #2
#5	("diabetes"):ti,ab,kw
#6	(mellitus):ti,ab,kw
#7	#2 AND #6
#8	#4 OR #3 OR #5 OR #7
#9	("adherence"):ti,ab,kw
#10	("compliance"):ti,ab,kw
#11	#9 OR #10
#12	("antidiabetic"):ti,ab,kw
#13	("drug"):ti,ab,kw
#14	(agent):ti,ab,kw
#15	#13 OR #14
#16	#12 AND #15
#17	(glucose):ti,ab,kw
#18	("lowering"):ti,ab,kw
#19	("antihyperglycemic"):ti,ab,kw
#20	#15 AND #17 AND #18
#21	#15 AND #19
#22	("tolbutamide"):ti,ab,kw
#23	("chlorpropamide"):ti,ab,kw
#24	("glibenclamide"):ti,ab,kw
#25	("glimepiride"):ti,ab,kw
#26	("glipizide"):ti,ab,kw
#27	("Gliclazide"):ti,ab,kw
#28	("gliquidone"):ti,ab,kw
#29	("repaglinide"):ti,ab,kw
#30	("nateglinide"):ti,ab,kw
#31	("mitiglinide"):ti,ab,kw
#32	("pioglitazone"):ti,ab,kw

#33 (semaglutide):ti,ab,kw
#34 (vildagliptin):ti,ab,kw
#35 ("sitagliptin"):ti,ab,kw
#36 (saxagliptin):ti,ab,kw
#37 (linagliptin):ti,ab,kw
#38 (alogliptin):ti,ab,kw
#39 ("metformin"):ti,ab,kw
#40 (dapagliflozin):ti,ab,kw
#41 (canagliflozin):ti,ab,kw
#42 (empagliflozin):ti,ab,kw
#43 ("sulphonylurea"):ti,ab,kw
#44 ("thiazolidinedione"):ti,ab,kw
#45 ("glitazone"):ti,ab,kw
#46 ("GLP-1"):ti,ab,kw AND ("receptor"):ti,ab,kw AND (agonist):ti,ab,kw
#47 ("DPP-IV"):ti,ab,kw AND ("inhibitor"):ti,ab,kw
#48 (gliptin):ti,ab,kw
#49 ("SGLT 2"):ti,ab,kw AND ("inhibitor"):ti,ab,kw
#50 #16 OR #20 OR #21 OR #22 OR #23 OR #24 OR #25 OR #26 OR #27 OR #28 OR #29 OR #30 OR #31 OR #32 OR #33 OR #34 OR #35 OR #36 OR #37 OR #38 OR #39 OR #40 OR #41 OR #42 OR #43 OR #44 OR #45 OR #46 OR #47 OR #48 OR #49
#51 #8 AND #11 AND #50

Table S1. Characteristics of studies included in the systematic review

Study name	Study design	Data collection	Age in years, mean ± SD (range)	Men, %	Continent	Study population	Definition of adherent patients	Included in the subgroup analysis
Abdullah, 2019	Cross-sectional	Questionnaire	(41-64)	46.6	Asia	Adult patients with T2D	Score ≥27 in the MCQ questionnaire	Yes (a,d)
Acharya, 2019	Cross-sectional	Questionnaire	49.8 ± 10.5	37.0	Asia	Adult patients with T2D	Score >8 in the MMAS-8 scale	Yes (a,d)
Adisa, 2013	Cross-sectional	Questionnaire	60.2 ± 10.2	38.6	Africa	Adult patients with T2D	Score <1 in the MMAPS scale	Yes (a,d)
Aditama, 2019	Cross-sectional	Questionnaire	45 (range unknown)	20.0	Asia	Adult patients with T2D	Mean score =1 in a 21-item questionnaire	Yes (a)
Afaya, 2020	Cross-sectional	Questionnaire	57.5 ± 11.8	31.8	Africa	Adult patients with T2D	Score 6-8 in the MAQ questionnaire	Yes (a,d)
Ahmad, 2013	Cross-sectional	Questionnaire	56.0 ± 9.1	36.8	Asia	Adult patients with T2D	Score > 27 in the MCQ questionnaire	Yes (a,d)
Aloudah, 2018	Cross-sectional	Questionnaire	57.8 ± 8.7	59.7	Asia	Adult patients with T2D	Score =8 in the MMAS-8 questionnaire	Yes (a,b)
AlQarni, 2019	Cross-sectional	Questionnaire	44.2 ± 15.6	67.0	Asia	Adult patients with T2D	Score 30-33 in the GMAS scale	Yes (a,b)
Aminde, 2019	Cross-sectional	Questionnaire	56.8 ± 2.7	29.9	Africa	Adult patients with T2D	Score ≥27 in the MCQ questionnaire	Yes (a,d)
An, 2014	Cohort study	Administrative data	58.7 ± 9.3	61.7	Asia	Adult patients with T2D	"MPR ≥90%, calculated as % of total days' supply of medication/number of days in the evaluation period"	Yes (a,d)
Andanalusia, 2019	Cross-sectional	Questionnaire	55.0 ± 9.8	28.3	Asia	Adult patients with diabetes	Score =12 in the ARMS scale	Yes (a)
Arulmozhi, 2014	Cross-sectional	Questionnaire	54.0 ± 12.0	50.0	Asia	Adult patients with T2D	Score =8 in the MMAS-8 questionnaire	Yes (a,d)
Ashur, 2015	Cross-sectional	Questionnaire	54.4 ± 10.0	41.1	Africa	Adult patients with T2D	Score ≥6 in the MMAS-8 questionnaire	Yes (a,d)
Ayoub, 2019	Cross-sectional	Questionnaire	59.2 ± 10.7	40.2	Asia	Adult patients with T2D	Score ≥38 in the LMAS-14 scale	Yes (a,f)
Balkhi, 2019	Cross-sectional	Administrative data	58.2 ± 10.8	37.7	Asia	Adult patients with T2D	"Modified MPR (mMPR) ≥80%, calculated as % of total days' supply for all OADs fills/number of days covered in the follow-up period"	Yes (a,b,f)
Basak, 2014	Cohort study	Administrative data	53.9 ± 6.7	55.5	America	Adult patients with T2D	"PDC ≥80%, calculated by proportion of days on medications out of all days until the end of each quarter of observation period"	Yes (a,d)
Bell, 2017	Cohort study	Administrative data	54.3 ± 9.6	53.7	America	Adult patients with T2D who had newly initiated treatment with an SGLT-2i or a sulfonylurea during the follow-up period (incident users)	"PDC ≥80%, calculated by dividing the number of days the patient was "covered" by the medication during the follow-up period by 180 days"	Yes (a,c,e)
Benzaravy, 2019	Cross-sectional	Questionnaire	56.4 ± 12.0	32.1	Asia	Adult patients with T2D	Score ≥6 in the MMAS-8 questionnaire	Yes (a)
Bloomgarden, 2017	Cohort study	Administrative data	56.3 ± 0.07	54.6	America	Adult patients with T2D	"PDC ≥ 80%, calculated as number of days with drug supplied divided for the follow-up period"	Yes (a,d)
Bonger, 2018	Cross-sectional	Questionnaire	51.1 ± 10.6	43.3	Africa	Adult patients with T2D	Percent score ≥60% in a structured and pretested questionnaire	Yes (a,d)
Bruce, 2015	Cross-sectional	Questionnaire	(18-75)	47.5	Africa	Adult patients with T2D	Score ≥3 in the MMAS-4 questionnaire	Yes (a)

Bryson, 2013	Cohort study	Administrative data	67.9 ± 11.0	97.9	America	Adult patients with diabetes	PDC ≥ 80%	Yes (a)
Buysman, 2017	Cohort study	Administrative data	55.6 ± 9.4	59.4	America	Adult patients with T2D without use of any SGLT-2 inhibitor 12 months prior to the index date (incident users)	"PDC ≥ 80%, calculated as the number of days that drug was available divided by the length of the follow-up period"	Yes (a,b,c,e)
Cai, 2016	Cohort study	Administrative data	54.3 ± 2.5	57.1	America	Adult patients with T2D whose first pharmacy claim for a newer OAD was during the inclusion period (incident users)	"PDC ≥ 80%, calculated as number of days covered by pharmacy claims for the index agents divided by 360 days"	Yes (a,c,e)
Cai, 2017	Cohort study	Administrative data	55.0 ± 9.1	56.0	America	Adult patients with T2D newly initiating SGLT-2 inhibitors, GLP-1 receptor agonists, or DPP-4 inhibitors during the inclusion period (incident users)	"PDC ≥ 80%, calculated as number of days the index drug was available or 'covered' divided by 360 days"	Yes (a,c,d,e)
Carls, 2017a	Cohort study	Administrative data	62.5 ± 0.7	58.0	America	Adult patients with T2D newly initiating antihyperglycemic drugs during the inclusion period (incident users)	PDC ≥ 80%	Yes (a,c,e)
Carls, 2017b	Cohort study	Administrative data	63.0 ± 0.5	54.8	America	Adult patients with T2D initiating OAD therapy during the inclusion period (incident users)	PDC ≥ 80%	Yes (a,c,e)
Chang, 2015	Cohort study	Administrative data	56.5 ± 11.6	52.1	Asia	Adult patients with T2D	"MPR ≥80%, calculated as the ratio of the number of days of prescribed medication divided by the total number of days in each study year"	Yes (a)
Chen, 2013	Cohort study	Administrative data	55.7 ± 11.3	52.5	Asia	Newly diagnosed adult patients with T2D initiating OAD therapy (incident users)	"MPR between 80% and 120%, calculated as ratio of the total days with a supply of a prescribed medication divided by follow-up period"	Yes (a,e)
Chen, 2016	Cohort study	Administrative data	55.6 ± 11.3	52.8	Asia	Adult patients with T2D without T2D-related claims over the 3 years before the index date (incident users)	"MPR ≥80%, calculated as ratio of the number of days of medications supplied divided by the total number of days in each study year"	Yes (a,e)
Cheng, 2013	Cohort study	Administrative data	55.6 ± 11.3	53.0	Asia	Adult patients with newly diagnosed T2D, without T2D-related claims over the 3 years before the index date (incident users)	"MPR ≥80%, calculated by dividing total number of days for which medication was prescribed by the number of days in a year"	Yes (a,e)
Chepulis, 2020	Cohort study	Administrative data	65.0 ± 8.6	55.5	Oceania	Patients with T2D	"MPR ≥80%, calculated by summing the days of medication supply from the first to the last prescription divided by total number of days of the prescription period"	Yes (a,b,c,d)
Chew, 2015	Cross-sectional	Questionnaire	56.9 ± 10.2	46.0	Asia	Adult patients with T2D	Score ≥6 in the MMAS-8 questionnaire	Yes (a,d)
Chong, 2014	Cohort study	Administrative data	57 (range unknown)	54.3	America	Patients with T2D	"PDC ≥80%, calculated as ratio between number of days' supply of medication in the index period and number of days in the study period"	Yes (a,c)
de Vries, 2014	Cross-sectional	Questionnaire	65.8 ± 9.5	55.3	Europe	Adult patients with T2D	Score >20 in the MARS scale	Yes (a)

Degefa, 2020	Cohort study	Verbal interview	51.7 ± 12.2	50.7	Africa	Adult patients with T2D	"Receiving at least 80% of antidiabetic medications as per prescribed frequency and dose in the last 7 days"	Yes (a)
Dossa, 2015	Cohort study	Administrative data	(55-74)	47.8	America	Adult patients with T2D who newly use OADs, without claims of an OAD in the 1-year period before the OAD initiation date (incident users)	"PDC ≥80%, calculated as total number of days covered by either any oral antidiabetic drug, divided by 365 days"	Yes (a)
Elsous, 2017	Cross-sectional	Questionnaire	56.4 ± 10.4	44.2	Asia	Patients with T2D	Score =4 in the MMAS-4 questionnaire	Yes (a,d)
Fai, 2017	Cross-sectional	Questionnaire	(35-64)	/	America	Adult patients with T2D	Score =8 in the MMAS-8 questionnaire	Yes (a)
Farmer, 2016	Cohort study	Administrative data	64.2 ± 0.7	63.8	Europe	Adult patients with T2D who were newly-prescribed metformin, sulfonylureas, thiazolidinediones or DPP-4i (incident users)	"MPR ≥80%, calculated by using prescription data from the date of the first prescription for that patient to the next prescription after 365 days from the first prescription date"	Yes (a,c,e)
Farr, 2014	Cohort study	Administrative data	55.9 ± 11.5	54.3	America	Adult patients with T2D without claims for OADs in the 12 months prior the index date (incident users)	"PDC ≥80%, calculated by taking the number of days a patient had the index drug class on hand during the 1-2-year follow-up based on the days' supply field on pharmacy claims divided by follow-up period"	Yes (a,c,e)
Farr, 2016	Cohort study	Administrative data	55.2 ± 9.8	53.0	America	Adult patients with T2D without claims for DPP-4i in the 12 months prior the index date (incident users)	"PDC ≥80%, calculated by taking the number of days a patient had the index drug class on hand divided by follow-up time"	Yes (a,c,e)
Garcia, 2019	Cohort study	Administrative data	55.2 ± 9.8	36.4	America	Adult patients with T2D	"PDC ≥80%, calculated as the sum of the days covered (based on fill date and days' supply) divided by days monitored"	Yes (a,b)
Gatwood, 2018a	Cohort study	Administrative data	62.7 ± 10.6	95.6	America	Adult patients with T2D who received an OAD as first-line therapy for the first time (incident users)	"PDC ≥80%, calculated as the sum of the days' supply within each person's records divided for 365 days"	Yes (a,b,c,d,e)
Gatwood, 2018b	Cohort study	Administrative data	62.2 ± 0.7	95.6	America	Adult patients with T2D who received an OAD as first-line therapy for the first time (incident users)	"PDC ≥80%, calculated as ratio (%) between the sum of days the medication was on hand and the number of days in the observation period"	Yes (a,b,c,e)
Gatwood, 2021	Cohort study	Administrative data	55.2 ± 11.4	52.9	America	Adult patients with T2D	"PDC ≥ 80%. The denominator was 180 days and the numerator was a count of the days in the observation period on which patients had medication in their possession according to the days' supply field"	Yes (a)
Gordon, 2018	Cohort study	Administrative data	57.6 ± 1.4	61.7	Europa	Adult patients with T2D	"MPR ≥80%, defined as the total number of days of available medication (calculated as the quantity of drug prescribed divided by the daily dose), divided by the length of the analysis period"	Yes (a,d)
Graetz, 2020	Cohort study	Administrative data	63.8 ± 13.0	53.8	America	Adult patients with diabetes	"PDC ≥80%, which was based on the number of days' supply of oral diabetes prescription drug dispensed in each month of the study"	Yes (a)
Guenette, 2013	Cohort study	Administrative data	-	48.4	America	Adult patients with T2D who were newly dispensed an OAD during the inclusion period, without prescriptions in the previous year (incident users)	"MPR ≥80%, calculated as the number of days' supply of OADs or insulin during the 365-day period after OADs initiation divided by 365 days"	Yes (a,b,e)

Guenette, 2015	Cross-sectional	Questionnaire	(39-78)	57.8	America	Adult patients with T2D	Score =4 in the MMAS-4 questionnaire	Yes (a)
Guenette, 2016	Cross-sectional	Questionnaire	62.7 ± 9.1	58.6	America	Adult patients with T2D	Score =8 in the MMAS-8 questionnaire	Yes (a,c)
Hagen, 2014	Cohort study	Administrative data	53.0 ± 7.5	85.2	America	Adult patients with diabetes	"PDC ≥80%, calculated as the number of days with drugs on hand divided by 365"	Yes (a,b)
Haines, 2018	Cross-sectional	Questionnaire	62.0 ± 8.8	53.6	America	Adult patients with T2D	Score ≥7 in the MMAS-8 questionnaire	Yes (a)
Hansen, 2015	Cohort study	Administrative data	65.0 ± 1.7	95.8	America	Adult patients with diabetes	"Refill adherence ≥ 80%, calculated with a claim-based continuous multiple-interval gap (CMG) that estimates the % of days a patient did not possess medication"	Yes (a,b)
Hayashi, 2018	Cross-sectional	Questionnaire	65.0 ± 11.0	64.7	Asia	Adult patients with T2D	-	Yes (a)
Hedna, 2013	Cross-sectional	Administrative data	68.3 ± 11.4	49.1	Europe	Adult patients with diabetes	"Refill adherence ≥80%, measured using the continuous measure of medication acquisition (CMA). CMA is the cumulative number of days' supply divided by the number of days during the study period"	Yes (a)
Heissam, 2015	Cross-sectional	Questionnaire	51.6 ± 10.8	55.6	Africa	Adult patients with T2D	Result ≥75% in the MTA scale	Yes (a,d)
Hong, 2014	Cohort study	Administrative data	55 (range unknown)	58.3	Asia	Newly diagnosed adult patients with T2D who received a new hypoglycaemic prescription during the inclusion period (incident users)	"MPR ≥80%, calculated as ratio between the total number of days covered in the supply of prescribed medication and the total number of days of study participation per participant"	Yes (a,e)
Horii, 2019	Cohort study	Administrative data	47.0 ± 8.1	90.2	Asia	Adult patients with T2D followed for 3 years after medication initiation (incident users)	"PDC ≥80%, calculated as number of days for which the antidiabetic agent was possessed during the observation period/observation period"	Yes (a,b,c,e)
Horsburgh, 2019	Cohort study	Administrative data	55 (range unknown)	52.8	Oceania	Adult patients with T2D who used metformin for the first time during the inclusion period (incident users)	"MPR ≥80%, defined as the proportion of days during a defined time period that a person was in possession of a medication, as determined by pharmacy dispensing records"	Yes (a,c,d,e)
Horvat, 2018	Cross-sectional	Pill counting	-	42.1	Europe	Adult patients with T2D	"90-105% of the prescribed medication taken. Adherence (%) was calculated using a pill count method, by dividing the difference in the number of pills recorded in the first home visit and the number of pills remaining in the package at the second home visit with the number of pills prescribed for the intervals"	Yes (a,b,d)
Huber, 2016	Cohort study	Administrative data	69.0 ± 11.9	53.7	Europe	Adult patients with diabetes	"PDC ≥80%, calculated as the number of days of medications supplied between the 1rst prescription (x, index date) and the last date (y) of a 1-year interval following the index date, divided by the total days of the interval"	Yes (a,c,d)
Ibrahim, 2021	Cross-sectional	Questionnaire	54.9 ± 15.3	45.4	Asia	Adult patients with T2D	Score >11 in a sub-version of the LMAS-14 questionnaire	Yes (a,f)
Iyengar, 2014	Cohort study	Administrative data	74.0 ± 6.5	42.8	America	Adult patients with diabetes	"PDC ≥80%, calculated as the total days' supply divided by 365 and multiplied by 100"	Yes (a)
Iyengar, 2016	Cohort study	Administrative data	73.8 ± 0.1	43.3	America	Adult patients with diabetes	"PDC ≥80%, calculated by taking the total days' supply divided by 365 days and multiplying the result by 100"	Yes (a)
Jamous, 2011	Cross-sectional	Questionnaire	56.3 ± 9.8	63.8	Asia	Adult patients with T2D	Score =8 in the MMAS-8 questionnaire	Yes (a,b,d)

Jannuzzi, 2014	Cross-sectional	Verbal interview	59.8 ± 13.9	35.3	America	Adult patients with T2D	“Appropriate use (≥80%) of the prescribed doses”	Yes (a)
Jannuzzi, 2020	Cohort study	Verbal interview	60.2 ± 10.9	34.3	America	Adult patients with T2D	“Appropriate use (≥80%) of the prescribed doses”	Yes (a)
Jarab, 2014	Cross-sectional	Questionnaire	64.0 ± 9.8	56.7	Asia	Adult patients with T2D	Score =4 in the MMAS-4 questionnaire	Yes (a,d)
Jiang, 2020	Cohort study	Administrative data	77 (range unknown)	45.9	Asia	Adult patients with T2D	“PDC ≥80%, calculated as ratio between the number of days covered by OAD prescription during the observation period and the observation period”	Yes (a,b)
Khan, 2014	Cross-sectional	Questionnaire	51.4 ± 12.2	44.0	Asia	Adult patients with T2D	Score =4 in the MMAS-4 questionnaire	Yes (a,b)
Kharjul, 2019	Cohort study	Administrative data	(21-100)	52.0	Oceania	Adult patients with T2D	“PDC ≥80%, calculated as total days’ supply divided by 356 days”	Yes (a,b)
Kim, 2016	Cross-sectional	Questionnaire	57.4 ± 12.3	38.8	Asia	Adult patients with T2D	Score =12 in the Korean version of the ARMS scale	Yes (a,f)
Kim, 2018	Cohort study	Administrative data	50 (range unknown)	61.2	Asia	Newly diagnosed T2D patients (incident users)	“PDC ≥80%, calculated from the patients’ prescription record”	Yes (a,b,c,e)
Koyanagi, 2016	Cross-sectional	Leftover drug counting	70.1 ± 11.3	60.7	Asia	Adult patients with diabetes	“Prescription reduction ratio (PRR) <0.2, calculated by evaluating the number of drugs originally prescribed and the reduced amount after use of leftover drugs”	Yes (a,c,d)
Kumar, 2019	Cross-sectional	Questionnaire	46.6 ± 9.3	54.8	Asia	Adult patients with T2D	Score between 0 and 2 in an eight-item questionnaire	Yes (a,c,d)
Kurtyka, 2016	Cohort study	Administrative data	51.1 ± 8.4	74.4	Asia	Adult patients with T2D who initiated a DPP-4i during the index period, without use of DPP-4i in the prior 12 months (incident users)	“PDC ≥80%, calculated based on the total number of days during follow-up with medication on hand within the first 12 months of follow-up divided by the follow-up time”	Yes (a,b,c,d,e)
Lee, 2013	Cross-sectional	Questionnaire	59.3 ± 11.2	61.5	Asia	Adult patients with T2D	Score =8 in the MMAS-8 questionnaire	Yes (a,b)
Lee, 2017	Cross-sectional	Questionnaire	62.0 ± 10.4	46.6	Asia	Adult patients with T2D	Score = 25 in the MARS scale	Yes (a,b,f)
Leporini, 2016	Cross-sectional	Questionnaire	(54-75)	67.1	Europe	Adult patients with T2D receiving first drug therapy or new diabetic treatment during the inclusion period (incident users)	Score =8 in the MMAS-8 questionnaire	Yes (a,d,e)
Lo-Ciganic, 2015	Cohort study	Administrative data	48.2 ± 10.0	33.5	America	Adult patients with T2D with first prescription fill for OADs during the inclusion period, without OADs prescriptions in the 6 months prior (incident users)	“PDC ≥80%, defined as the number of days covered with oral antidiabetics divided by the number of days plus the accumulated days supplied from the last prescription”	Yes (a,e)
Lo-Ciganic, 2016	Cohort study	Administrative data	45.5 ± 11.2	32.2	America	Adult patients with T2D with first prescription fill for OADs during the inclusion period, without OADs prescriptions in the 6 months prior (incident users)	“PDC ≥80%, calculated monthly with ≥1 oral antidiabetic medication, given that 95 % of OAD claims were 30-day prescriptions”	Yes (a,b,d,e,f)
Lokhandwala, 2016	Cohort study	Administrative data	54.9 ± 11.2	62.4	America	Adult patients with T2D naïve to OADs during the pre-index period (incident users)	“MPR ≥80%, calculated as the sum of the days’ supply for all index regimen medication fills during the follow-up period divided by follow-up”	Yes (a,d,e)

Lopez-Simarro, 2016	Cohort study	Mixed method (verbal interview and administrative data)	67.5 ± 10.7	53.4	Europe	Adult patients with T2D	"Mixed-method between self-reported adherence (Haynes–Sackett test) and medication refill ≥80%, determined from the ratio (%) between the total doses refilled from the pharmacy office in the previous year and the total doses prescribed according to the case history"	Yes (a)
Mallah, 2019	Cross-sectional	Questionnaire	58.0 ± 11.7	43.0	Asia	Adult patients with T2D	Score =7 in the DMAS-7 tool	Yes (a,f)
Manteuffel, 2014	Cohort study	Administrative data	41 (range unknown)	58.9	America	Adult patients with diabetes	"MPR ≥80%, defined as the number of days of medication supply divided by the number of days between the first and last medication refill dates"	Yes (a,b)
Martinez-Perez, 2021	Cross-sectional	Questionnaire	65.4 ± 9.7	58.5	Europe	Adult patients with T2D	Score =8 in the MMAS-8 questionnaire	Yes (a)
Mhatre, 2016	Cohort study	Administrative data	74.8 (range unknown)	43.7	America	Adult patients with diabetes	PDC ≥ 80%	Yes (a)
Miller, 2015	Cohort study	Administrative data	50.7 ± 3.2	49.2	America	Adult patients with diabetes	"MPR ≥ 80%, calculated as days supplied divided by the 365 days in a year, less any days spent in the hospital"	Yes (a)
Min, 2019	Cohort study	Administrative data	(57-72)	96.0	America	Adult patients with T2D newly using metformin, without OAD refill in the prior 180 days (incident users)	"PDC ≥80%, calculated after 1 year following intensification with a sulfonylurea"	Yes (a,b,c,d,e)
Mirahmadizadeh, 2020	Cross-sectional	Questionnaire	56.9 ± 0.52	41.2	Asia	Adult patients with T2D	Score =8 in the MMAS-8 questionnaire	(a,d)
Mroueh, 2018	Cross-sectional	Questionnaire	59.3 ± 10.8	45.7	Asia	Adult patients with T2D	Score >38 in the LMAS-14 questionnaire	Yes (a,f)
Mukherjee, 2013	Cross-sectional	Verbal interview	42.6 ± 15.7	66.4	Asia	Adult patients with T2D	"Taking more than 80% of the prescribed anti-diabetes medicines and having HbA1C of <7%"	Yes (a,d)
Naffaa, 2020	Cohort study	Administrative data	57.5 ± 13.1	52.0	Asia	Adult patients with T2D who initiated therapy with metformin during the inclusion period (incident users)	"PDC ≥80%, calculated by dividing the supply days of dispensed metformin by the total follow-up time (including the day when metformin was last dispensed)"	Yes (a,b,c)
Nichols, 2016	Cohort study	Administrative data	58.6 (range unknown)	9.1	America	Adult patients with T2D who initiated therapy with metformin as their first ever OAD during the inclusion period (incident users)	"BRBPDC ≥80%. This method estimates adherence over the 90 days preceding a given HbA1c measurement"	Yes (a,c,e)
Nichols, 2018	Cohort study	Administrative data	-	52.0	America	Adult patients with T2D	"PDC ≥80%, defined as the % of days in the measurement period covered by prescription fills of the same medication or same therapeutic class"	Yes (a,f)
Nigam, 2012	Cohort study	Administrative data	54.2 ± 0.1	57.6	America	Adult patients with T2D	"MPR ≥75%, calculated as the sum of days' supply for each prescription claim in the post-index period expressed as a % of 365 days"	Yes (a,c)
Nishimura, 2019a	Cohort study	Administrative data	59.9 ± 8.0	64.8	Asia	Adult patients without a prescription for any antidiabetic drug of interest during the 12-month pre-index period (incident users)	"PDC ≥80%, calculated from the index date to the first discontinuation of index treatment. Adherence was calculated according to the number of antidiabetic drug prescription days"	Yes (a,c,e)
Nishimura, 2019b	Cohort study	Administrative data	59.9 ± 9.3	72.3	Asia	Adult patients with T2D	"PDC ≥80%, calculated as the number of days the treatment was available to the patient divided by the number of days in the treatment period"	Yes (a,d)

Ofori-Asenso, 2019a	Cohort study	Administrative data	62.7 ± 13.3	57.2	Oceania	Adult patients with T2D who were newly dispensed DPP-4i, without prescriptions for DPP-4i in the prior 12 months (incident users)	"PDC ≥80%, calculated by dividing the number of days the patient was covered by medication during the follow-up period by 365 days"	Yes (a,c,e)
Ofori-Asenso, 2019b	Cohort study	Administrative data	60.9 ± 11.5	59.5	Oceania	Adult patients with T2D who were newly dispensed SGLT-2i, without prescriptions for SGLT-2i in the prior 12 months (incident users)	"PDC ≥80%, calculated by dividing the number of days the patient was covered by medication during the follow-up period by 365 days"	Yes (a,c,e)
Oh, 2020	Cohort study	Administrative data	68.4 ± 12.0	60.9	Asia	Treatment naïve adult patients with T2D (incident users)	"PDC ≥80%, calculated as the total number of days the DPP-4i was taken divided by the total number of days in the treatment period in which treatment was expected to take"	Yes (a,c,e)
Olickal, 2021	Cross-sectional	Questionnaire	53 (range unknown)	45.8	Asia	Adult patients with T2D	Score =0 in the MGL scale	Yes (a)
O'Shea, 2013	Cohort study	Administrative data	-	44.0	Europe	Adult patients with T2D who were dispensed either a sulfonylurea or metformin during the inclusion period, without prescription claims for any OADs in the previous 6 months (incident users)	"MPR ≥80%, calculated as the number of days of medication obtained by a patients during a specific period, divided by the number of days in the specified period"	Yes (a,b,c,d,e)
O'Shea, 2015	Cohort study	Administrative data	-	51.4	Europe	Adult patients with T2D who were dispensed either a sulfonylurea or metformin during the inclusion period, without prescription claims for any OADs in the previous 6 months (incident users)	"MPR ≥80%, calculated as the number of days of medication obtained by a patient during a specific period, divided by the number of days in the specified period"	Yes (a,d,e)
Oung, 2017	Cohort study	Administrative data	52.0 ± 10.0	40.8	America	Adult patients with diabetes	"PDC ≥80%, defined as the number of days the medication is supplied during the study period divided by the number of days in the study period"	Yes (a,c)
Pascal, 2012	Cross-sectional	Questionnaire	36.8 ± 5.4	37.5	Africa	Adult patients with T2D	Score =4 in a questionnaire using an ordinal scoring system of 0-4 points	Yes (a,f)
Peeters, 2015	Cross-sectional	Questionnaire	(30-69)	42.9	Asia/Europe	Adult patients with T2D	"Taking >80% of OADs, calculated using the MGL questionnaire"	Yes (a)
Quilliam, 2013	Cohort study	Administrative data	52.8 ± 1.4	51.1	America	Adult patients with T2D initiating treatment with metformin, a sulfonylurea, or a thiazolidinedione (incident users)	"MPR ≥80%, calculated by dividing the summed days' supply of the identified medications divided by the number of days between the first and last identified claims"	Yes (a,b,c,d,e)
Rahmatullah, 2021	Cross-sectional	Questionnaire	52.7 ± 11.2	52.7	Asia	Adult patients with T2D	-	Yes (a)
Rana, 2017	Cross-sectional	Questionnaire	50.2 ± 8.5	37.0	Asia	Adult patients with T2D	Score =8 in the MMAS-8 questionnaire	Yes (a,b)
Ranashinge, 2018	Cross-sectional	Questionnaire	60.6 ± 11.1	46.1	Asia	Adult patients with diabetes	No positive response in all three screens of the BMQ questionnaire	Yes (a,c)
Rascati, 2017	Cohort study	Administrative data	63.2 ± 8.4	49.2	America	Adult diabetic patients	"PDC ≥80%, calculated as the proportion of days during the 365-day post-index period that the patient has any DPP-4i on hand"	Yes (a,c)

Rathish, 2019	Cross-sectional	Questionnaire	(49-67)	50.0	Asia	Adult patients with T2D	Score =4 in the MMAS-4 questionnaire	Yes (a)
Reach, 2011	Cross-sectional	Questionnaire	59.0 ± 4.0	60.0	Europe	Adult patients with T2D	Score >3 in a 5-item questionnaire on adherence	Yes (a)
Reach, 2018	Cross-sectional	Questionnaire	67.9 ± 9.7	55.8	Europe	Adult patients with T2D	Score <1 in the six-item questionnaire by Girerd et al.	Yes (a)
Rinne, 2015	Cohort study	Administrative data	67.2 ± 3.5	97.9	America	Adult patients with diabetes	MPR ≥80%	Yes (a)
Romagnoli, 2021	Cohort study	Administrative data	(11-103)	52.0	Europe	Adult patients with T2D	"Ratio between Received Daily Dose (RDD) and Prescribed Daily Dose (PDD) ≥ 80%"	Yes (a,c,d)
Sacks, 2013	Cohort study	Administrative data	74.5 ± 6.4	35.0	America	Adult patients with diabetes	"MPR ≥80%, calculated as the days on which patients had medication available in each OAD class as the sum of days supplied for each fill over the calendar year, plus any days remaining from previous year fills"	Yes (a,c)
Sacks, 2015	Cohort study	Administrative data	73.8 ± 0.1	37.6	America	Adult patients with diabetes	"PDC ≥80%, calculated the PDC as the ratio of days supplied to calendar days in the observation period"	Yes (a)
Sankar, 2015	Cross-sectional	Questionnaire	54.2 ± 11.5	42.2	Asia	Adult patients with diabetes	Score ≥6 in the MMAS-8 questionnaire	Yes (a)
Saraiva, 2020	Cross-sectional	Questionnaire	(52-68)	35.7	America	Adult patients with T2D	Score =4 in the MMAS-4 questionnaire	Yes (a,c,d)
Satirapoj, 2020	Cross-sectional	Questionnaire	65.5 ± 10.0	49.3	Asia	Adult patients with T2D	"Taking the medication exactly as prescribed, self-reported on the 5-level Likert Scale"	Yes (a)
Sattler, 2014	Cohort study	Administrative data	74.2 ± 7.8	22.6	America	Adult patients with T2D	"PDC ≥80%, defined as the number of days of medication supply in the observation period multiplied per 100"	Yes (a,b)
Saundankar, 2016	Cohort study	Administrative data	71.6 ± 0.7	45.6	America	Adult patients with T2D	"PDC ≥80%, defined as the percent of days in the measurement period covered by prescription claims for the same medication"	Yes (a,b,c,f)
Schwab, 2019	Cohort study	Administrative data	(19-89)	51.6	America	Adult patients with T2D new to OADs entirely or initiating an OAD that the individual had not previously used (incident users)	"PDC ≥80%, defined as the number of days with an OAD on-hand divided by the number of days from the index date to the end of the measurement period"	Yes (a,e)
Shields, 2020	Cohort study	Administrative data	-	/	Europe	Adult patients with T2D	"MPR ≥80%, defined as the number of days of available medication (calculated by dividing the quantity prescribed by the daily dose) divided by the number of days between the first prescription and the adherence period end date (365 days), multiplied per 100"	Yes (a,d)
Shrestha, 2013	Cross-sectional	Questionnaire	58.1 ± 11.6	48.0	Asia	Adult patients with T2D	-	Yes (a,f)
Sicras-Mainar, 2014	Cohort study	Administrative data	74.2 ± 6.2	49.1	Europe	Adult patients with T2D	"Compliance ≥80%, calculated by dividing the total number of tablets dispensed by those recommended or prescribed"	Yes (a,d)
Simard, 2015	Cohort study	Administrative data	67.0 ± 10.0	49.0	America	Adult patients with diabetes without an OAD or insulin prescription within the 2 years prior to the cohort entry date (incident users)	"MPR ≥80%, calculated as the number of days of medication supply over a given follow-up period, expressed as percentage"	Yes (a,e)
Simard, 2018	Cohort study	Administrative data	72.3 ± 8.7	55.4	America	Adult patients with diabetes without metformin, an OAD or insulin prescription within the 2 years prior to the	"MPR ≥80%, defined as the proportion of days medication supplied over a given follow-up period, expressed as percentage"	Yes (a,c)

						cohort entry date (incident users)		
Simon-Tuval, 2018	Cross-sectional	Administrative data	55.3 ± 9.6	42.9	Asia	Adult patients with T2D	"PDC ≥80%, calculated by dividing the number of days the medication was available by the total time interval from index date through the month of the last prescription refill within the follow-up period"	Yes (a)
Singhal, 2019	Cohort study	Administrative data	53.4 ± 9.1	50.7	America	Adult patients with T2D	"PDC ≥80%, calculated as the total number of days the index medication was available divided by the total number of days in the follow-up period"	Yes (a,c)
Taira, 2017	Cohort study	Administrative data	67.8 ± 2.6	52.7	America	Adult patients with T2D	"MPR ≥80%, calculated as the ratio of the number of days for which a patient has medication on hand divided by the total number of days a patient was enrolled in a drug plan"	Yes (a)
Tanabe, 2017	Cohort study	Administrative data	65.5 ± 3.4	62.6	Asia	Adult patients with T2D	"Total sum of prescription days/total number of observation days ≥ 80%"	Yes (a,c)
Tandon, 2015	Cross-sectional	Questionnaire	57.5 ± 10.0	27.0	Africa	Adult patients with T2D	Score =8 in the MMAS-8 questionnaire	Yes (a,d)
Tang, 2020	Cohort study	Administrative data	66.4 ± 12.3	58.6	Europe	Adult patients with T2D without metformin use in the 12 months prior the first prescription in the index year (incident users)	"PDC ≥80%, calculated as the sum of days' supply of metformin divided by the number of in the follow-up period"	Yes (a,c,e)
Tiv, 2012	Cross-sectional	Questionnaire	65.0 ± 11.1	54.4	Europe	Adult patients with T2D	Answering "no" to all questions of a six-item questionnaire	Yes (a)
Tunceli, 2015a	Cohort study	Administrative data	60.7 ± 12.6	50.6	America	Adult patients with T2D	"PDC ≥80%, calculated as the number of days with the drug on-hand divided follow-up period"	Yes (a,d)
Tunceli, 2015b	Cohort study	Administrative data	-	57.0	Europe	Adult patients with T2D	"PDC ≥80%, calculated as the number of days with drug supplied during the observation period divided follow-up period"	Yes (a,b,d)
Vichayanrat, 2013	Cohort study	Verbal interview	52.0 ± 11.7	46.7	Asia	Adult patients with T2D either newly diagnosed starting OADs or treated with OAD monotherapy for less than 6 months (incident users)	"Compliance ≥ 90%"	Yes (a,c,e)
Vietri, 2016	Cross-sectional	Questionnaire	58.9 ± 12.2	55.0	America	Adult patients with T2D	"Adherence has been calculated with the adherence estimator, a three-item measure which allows for categorization of patients into low, medium, or high risk for non-adherence"	Yes (a)
Vittorino Gaddi, 2014	Cohort study	Administrative data	70 (range unknown)	50.9	Europe	Adult patients with diabetes who were first prescribed OADs during the recruitment period (incident users)	"MPR ≥80%, calculated as sum of total days' supply for all fills divided by number of days between the first and last fills plus days' supply of the last fill"	Yes (a,d,e)
Vlacho, 2021	Cohort study	Administrative data	60.8 ± 11.7	-	Europe	Adult patients with T2D initiating a combination therapy	"MPR≥ 80%, calculated as the number of days covered by dispensation divided by the number of days covered by prescription, which is defined as days between the date of initiation of index medication and discontinuation event or up to 24 months"	Yes (a,d)
Waari, 2018	Cross-sectional	Questionnaire	56.6 ± 11.9	32.4	Africa	Adult patients with T2D	Score =8 in the MMAS-8 questionnaire	Yes (a,d)
Walz, 2014	Cross-sectional	Questionnaire	69.0 ± 9.5	60.7	Europe	Adult patients with T2D	"Always taking medications as prescribed", assessed by a questionnaire developed by Grant et al.	Yes (a)

White, 2012	Cross-sectional	Pill counting	(62-74)	66.7	Europe	Adult patients with T2D	"90-100% of prescribed doses taken, measured by MEMS (Medication Event Monitoring System)"	Yes (a,b,c)
Wong, 2011	Cohort study	Administrative data	/	55.0	Asia	Adult patients with diabetes	"MPR ≥80%, defined as the ratio of total days of medication supplied (not including the last prescription) to total days in a period of time"	Yes (a,b,c)
Wong, 2012	Cohort study	Administrative data	67.9 ± 11.0	97.9	America	Adult patients with diabetes	"Having medications available for at least 80% of the period", measured with the validated ReComp algorithm	Yes (a)
Wong, 2014	Cohort study	Administrative data	67.8 ± 11.3	97.9	America	Adult patients with diabetes	"MPR ≥80%, which measure that estimates the proportion of days within an interval for which a patient has medication available"	Yes (a,b,f)
Wong, 2015	Cross-sectional	Questionnaire	63.2 ± 9.7	46.5	Asia	Adult patients with T2D	Score >6 in the MMAS-8 questionnaire	Yes (a,b,f)
Wu, 2016	Cross-sectional	Questionnaire	(34-83)	50.8	Asia	Adult patients with T2D	Score 6-8 in the MMAS-8 questionnaire	Yes (a)
Zhou, 2013	Cross-sectional	Verbal interview	53.1 ± 14.6	52.0	Asia	Adult patients with diabetes	"Taking ≥90% of the daily prescribed medication"	Yes (a)
Zhu, 2015	Cohort study	Administrative data	63.7 ± 4.7	50.9	Asia	Adult patients with T2D	"PDC ≥80%, defined as the total number of medication-covered days divided by 365 days"	Yes (a,b,d)
Zomahoun, 2016	Cohort study	Administrative data	62.6 ± 9.2	57.7	America	Adult patients with T2D	"PDC ≥80%, measured as the proportion of days covered using a pharmacy claims database"	Yes (a,b)
Zongo, 2016	Cross-sectional	Questionnaire	(59-68)	62.1	America	Adult patients with T2D	Score =8 in the MMAS-8 questionnaire	Yes (a,f)

List of abbreviations: ARMS: Adherence to Refills and Medication Scale; BMQ: Brief Medication Questionnaire; BRBPDC: Biologic Response Based Proportion of Days Covered; DMAS: Diabetes Medication Adherence Scale; DPP4-i = dipeptidyl peptidase-4 inhibitors; GMAS: General Medication Adherence Scale; LMAS-14: 14-item Lebanese Medication Adherence Scale; MARS: Medication Adherence Report Scale; MAQ: Medication Adherence Questionnaire; MCQ: Medication Compliance Questionnaire Adherence; MGL: Morisky, Green, and Levine; MMAPS: Morisky Adherence Predictor Scale; MMAS-4: four-item Morisky Medication Adherence Scale; MMAS-8: eight-item Morisky Medication Adherence Scale; MPR: Medication Possession Ratio; MTA: Measure Treatment Adherence Scale; PDC: Proportion of Days Covered; SGLT-2i = sodium-glucose co-transporter-2 inhibitors

*Stratification for: [a] age; [b] gender; [c] therapeutic class; [d] treatment regimen (monotherapy, dual therapy, oral therapy + insulin); [e] follow-up period for incident patients; [f] association between adherence and blood glucose control.

Table S2. Risk of bias of cross-sectional studies

**Study name**	Was the study's target population a close representation of the national population in relation to relevant variables?	Was the sampling frame a true or close representation of the target population?	Was some form of random selection used to select the sample, or was a census undertaken?	Was the likelihood of nonresponse bias minimal?	Were data collected directly from the subjects (as opposed to a proxy)?	Was an acceptable case definition used in the study?	Was the study instrument that measured the parameter of interest shown to have validity and reliability?	Was the same mode of data collection used for all subjects?	Were the numerator(s) and denominator(s) for the parameter of interest appropriate?	Total
Abdullah, 2019	1	1	1	1	1	1	1	1	0	8/9
Acharya, 2019	1	0	0	1	1	1	1	1	1	7/9
Adisa, 2013	0	1	1	1	1	1	1	1	0	7/9
Aditama, 2019	1	0	0	1	1	1	0	1	0	5/9
Afaya, 2020	0	0	0	1	1	1	1	1	1	6/9
Ahmad, 2013	0	0	1	1	1	1	1	1	1	7/9
Aloudah, 2018	1	1	0	1	1	1	1	1	1	8/9
AlQarni, 2019	1	0	1	1	1	1	1	1	1	8/9
Aminde, 2019	1	0	0	1	1	1	1	1	0	6/9
Andanalusia, 2019	1	0	0	1	1	1	1	1	0	6/9
Arulmozhi, 2014	1	1	1	1	1	1	1	1	0	8/9
Ashur, 2015	1	1	1	1	1	0	1	1	1	8/9
Ayoub, 2019	1	1	1	1	1	1	1	1	1	9/9
Benzaravy, 2019	1	0	0	1	1	0	1	1	1	6/9
Bonger, 2018	1	1	0	1	1	0	0	1	1	6/9
Bruce, 2015	0	1	0	1	1	1	1	1	1	7/9
Chew, 2015	0	1	1	1	1	0	1	1	1	7/9
de Vries, 2014	0	0	1	1	1	1	1	1	1	7/9
Elsous, 2017	0	1	1	1	1	1	1	1	1	8/9
Fai, 2017	0	0	0	1	1	1	1	1	0	5/9
Guenette, 2015	1	1	0	1	1	1	1	1	0	7/9
Guenette, 2016	1	1	1	1	1	1	1	1	1	9/9
Haines, 2018	0	1	1	1	1	1	1	1	0	7/9
Hayashi, 2018	0	0	1	1	1	0	0	1	1	5/9
Heissam, 2015	1	0	1	1	1	1	1	1	1	8/9
Ibrahim, 2021	1	1	1	1	1	1	0	1	1	8/9
Jamous, 2011	0	0	0	1	1	1	1	1	0	5/9
Jannuzzi, 2014	1	0	0	1	1	1	1	1	0	6/9
Jannuzzi, 2020	1	0	0	1	1	1	1	1	1	7/9
Jarab, 2014	0	1	1	1	1	1	1	1	0	7/9
Khan, 2014	0	1	1	1	1	1	1	1	1	8/9
Kim, 2016	0	1	1	1	1	1	1	1	1	8/9
Kumar, 2019	0	0	1	1	1	0	0	1	1	5/9
Lee, 2013	0	1	1	1	1	1	1	1	1	8/9
Lee, 2017	0	1	1	1	1	1	1	1	1	8/9
Leporini, 2016	0	0	1	1	1	1	1	1	1	7/9
Mallah, 2019	1	1	1	1	1	1	1	1	1	9/9
Martinez-Perez, 2021	0	1	1	1	1	1	1	1	1	8/9
Mirahmadizadeh, 2020	0	1	1	1	1	1	1	1	1	8/9
Mroueh, 2018	1	0	1	1	1	1	1	1	1	8/9
Mukherjee, 2013	0	0	1	1	1	1	0	1	1	6/9
Olickal, 2021	0	0	1	1	1	1	1	1	1	7/9

Pascal, 2012	0	0	1	1	1	1	0	1	0	5/9
Peeters, 2015	1	0	0	1	1	0	1	1	0	5/9
Rahmatullah, 2021	1	0	1	1	1	0	0	1	1	6/9
Rana, 2017	0	0	0	1	1	1	1	1	0	5/9
Ranashinge, 2018	1	0	1	1	1	1	1	1	1	8/9
Rathish, 2019	1	1	0	1	1	1	1	1	1	8/9
Reach, 2011	0	0	1	1	1	1	0	1	0	5/9
Reach, 2018	0	1	1	1	1	1	0	1	1	7/9
Sankar, 2015	1	1	1	1	1	0	1	1	1	8/9
Saraiva, 2020	1	0	0	1	1	1	1	1	1	7/9
Satirapoj, 2020	1	1	1	1	1	0	1	1	1	8/9
Shrestha, 2013	0	1	0	1	1	0	0	1	0	4/9
Tandon, 2015	1	0	0	1	1	1	1	1	0	6/9
Tiv, 2012	0	1	1	1	1	1	0	1	1	7/9
Vietri, 2016	1	1	1	1	1	1	1	1	1	9/9
Waari, 2018	1	0	1	1	1	1	1	1	0	7/9
Walz, 2014	0	1	1	1	1	0	0	1	1	6/9
Wong, 2015	0	1	1	1	1	0	1	1	1	7/9
Wu, 2016	1	1	1	1	1	0	1	1	0	7/9
Zhou, 2013	1	0	1	1	1	0	0	1	0	5/9
Zongo, 2016	1	0	1	1	1	1	1	1	1	8/9

Table S3. Risk of bias of cohort studies

**Study name**	Was the study's target population a close representation of the national population in relation to relevant variables?	Was the sampling frame a true or close representation of the target population?	Was some form of random selection used to select the sample, or was a census undertaken?	Was an acceptable case definition used in the study?	Was the study instrument that measured the parameter of interest shown to have validity and reliability?	Was the same mode of data collection used for all subjects?	Was the length of the shortest prevalence period for the parameter of interest appropriate?	Were the numerator(s) and denominator(s) for the parameter of interest appropriate?	Total
An, 2014	0	1	1	0	1	1	1	1	6/8
Balkhi, 2019	1	1	1	1	0	1	1	1	7/8
Basak, 2014	0	1	1	1	1	1	1	1	6/8
Bell, 2017	0	1	1	1	1	1	1	1	7/8
Bloomgarden, 2017	0	1	1	1	1	1	1	1	7/8
Bryson, 2013	0	0	0	0	1	1	0	1	3/8
Buysman, 2017	0	1	0	1	1	1	1	1	6/8
Cai, 2016	0	1	1	1	1	1	1	1	7/8
Cai, 2017	0	1	1	1	1	1	1	1	7/8
Carls, 2017a	0	1	0	0	1	1	1	1	5/8
Carls, 2017b	0	1	0	0	1	1	1	1	5/8
Chang, 2015	0	1	0	1	1	1	1	1	6/8
Chen, 2013	0	1	0	0	0	1	1	1	4/8
Chen, 2016	0	1	0	1	1	1	1	1	6/8
Cheng, 2013	0	1	1	1	1	1	1	1	7/8
Chepulis, 2020	1	1	1	1	1	1	1	1	8/8
Chong, 2014	0	1	0	1	1	1	1	1	6/8
Degefa, 2020	0	1	0	1	0	1	0	0	3/8
Dossa, 2015	0	1	0	1	1	1	1	1	6/8
Farmer, 2016	0	1	1	1	1	1	1	1	7/8
Farr, 2014	0	1	1	1	1	1	1	1	7/8
Farr, 2016	0	1	1	1	1	1	1	1	7/8
Garcia, 2019	0	1	1	1	1	1	1	1	7/8
Gatwood, 2018a	0	0	1	1	1	1	1	1	6/8
Gatwood, 2018b	0	0	1	1	1	1	1	1	6/8
Gatwood, 2021	0	1	1	1	1	1	1	1	7/8
Gordon, 2018	0	0	1	1	1	1	1	1	6/8
Graetz, 2020	1	1	1	1	1	1	0	1	7/8
Guenette, 2013	0	0	1	1	1	1	1	1	6/8
Hagen, 2014	0	0	1	1	1	1	1	1	6/8
Hansen, 2015	0	0	0	1	1	1	1	1	5/8
Hedna, 2013	1	1	1	1	1	1	1	1	8/8
Hong, 2014	0	1	0	1	1	1	1	1	6/8
Horii, 2019	0	0	1	1	1	1	1	1	6/8
Horsburgh, 2019	0	1	1	1	1	1	1	1	7/8
Horvat, 2018	0	1	0	0	1	1	0	1	4/8
Huber, 2016	1	1	1	1	1	1	1	1	8/8
Iyengar, 2014	0	1	1	1	1	1	1	1	7/8
Iyengar, 2016	0	1	1	1	1	1	1	1	7/8
Jiang, 2020	0	1	1	1	1	1	0	1	6/8
Kharjul, 2019	1	1	1	1	1	1	1	1	8/8

Kim, 2018	0	1	1	0	1	1	1	1	6/8
Koyanagi, 2016	1	1	1	1	1	1	0	1	7/8
Kurtyka, 2016	0	0	0	1	1	1	1	1	5/8
Lo-Ciganic, 2015	0	0	1	1	1	1	1	1	6/8
Lo-Ciganic, 2016	0	0	1	1	1	1	1	1	6/8
Lokhandwala, 2016	0	0	1	1	1	1	1	1	6/8
Lopez-Simarro, 2016	1	1	0	1	0	1	0	1	5/8
Manteuffel, 2014	0	1	1	1	1	1	1	1	7/8
Mhatre, 2016	0	1	1	0	1	1	1	1	6/8
Miller, 2015	0	1	1	1	1	1	1	1	7/8
Min, 2019	0	0	1	0	1	1	1	1	5/8
Naffaa, 2020	0	1	1	1	1	1	1	1	7/8
Nichols, 2016	0	1	0	1	0	1	1	1	5/8
Nichols, 2018	0	1	0	1	1	1	1	1	6/8
Nigam, 2012	1	1	1	1	0	1	1	1	7/8
Nishimura, 2019a	0	0	1	1	1	1	1	1	6/8
Nishimura, 2019b	1	0	1	1	1	1	1	1	7/8
Ofori-Asenso, 2019a	0	1	1	1	1	1	1	1	7/8
Ofori-Asenso, 2019b	0	1	1	1	1	1	1	1	7/8
Oh, 2020	0	1	1	1	1	1	1	1	7/8
O’Shea, 2013	0	1	1	1	1	1	1	1	7/8
O’Shea, 2015	0	1	1	1	1	1	1	1	7/8
Oung, 2017	0	1	1	1	1	1	1	1	7/8
Quilliam, 2013	0	1	1	1	1	1	1	1	7/8
Rascati, 2017	1	1	1	1	1	1	1	1	8/8
Rinne, 2015	0	0	1	1	0	1	0	1	4/8
Romagnoli, 2021	1	1	1	1	0	1	1	1	7/8
Sacks, 2013	0	0	0	1	1	1	1	1	5/8
Sacks, 2015	0	0	1	1	1	1	1	1	6/8
Sattler, 2014	0	0	1	1	1	1	0	1	5/8
Saundankar, 2016	0	1	1	1	1	1	1	1	7/8
Schwab, 2019	0	1	0	1	1	1	1	1	6/8
Shields, 2020	0	1	1	1	1	1	1	1	7/8
Sicras-Mainar, 2014	0	0	1	1	0	1	1	1	5/8
Simard, 2015	0	1	1	1	1	1	1	1	7/8
Simard, 2018	0	1	1	1	1	1	0	1	6/8
Simon-Tuval, 2018	0	1	0	1	1	1	0	1	5/8
Singhal, 2019	0	1	1	1	1	1	1	1	7/8
Taira, 2017	1	1	1	1	1	1	1	1	8/8
Tanabe, 2017	0	1	0	1	0	1	1	1	5/8
Tang, 2020	0	1	1	1	1	1	1	1	7/8
Tunceli, 2015a	0	1	1	1	1	1	1	1	7/8
Tunceli, 2015b	1	1	1	1	1	1	1	1	8/8
Vichayanrat, 2013	1	1	1	0	0	1	1	1	6/8
Vittorino Gaddi, 2014	0	0	1	1	1	1	1	1	6/8
Vlacho, 2021	0	0	1	1	1	1	1	1	6/8
White, 2012	1	1	1	0	1	1	0	1	6/8
Wong, 2011	1	1	1	1	1	1	0	1	7/8
Wong, 2012	1	0	1	1	1	1	0	1	6/8
Wong, 2014	1	0	1	1	1	1	1	1	7/8
Zhu, 2015	1	1	1	1	1	1	1	1	8/8
Zomahoun, 2016	1	1	0	1	1	1	0	1	6/8

Figure S1. Forest plot of proportions of adherent patients towards OADs and relative confidence intervals.

Source	Events	Total	Proportion	95% CI
Abdullah, 2019	67	117	0.57	[0.48; 0.66]
Acharya, 2019	50	161	0.31	[0.24; 0.39]
Adisa, 2013	60	130	0.46	[0.37; 0.55]
Aditama, 2019	8	40	0.20	[0.09; 0.36]
Afaya, 2020	209	247	0.85	[0.80; 0.89]
Ahmad, 2013	245	473	0.52	[0.47; 0.56]
Aloudah, 2018	158	395	0.40	[0.35; 0.45]
AlQarni, 2019	76	212	0.36	[0.29; 0.43]
Aminde, 2019	69	134	0.51	[0.43; 0.60]
An, 2014	388	492	0.79	[0.75; 0.82]
Andanalusia, 2019	5	60	0.08	[0.03; 0.18]
Arulmozhi, 2014	61	116	0.53	[0.43; 0.62]
Ashur, 2015	126	199	0.63	[0.56; 0.70]
Ayoub, 2019	196	500	0.39	[0.35; 0.44]
Balkhi, 2019	2652	5457	0.49	[0.47; 0.50]
Basak, 2014	3006	6043	0.50	[0.48; 0.51]
Bell, 2017	15746	27314	0.58	[0.57; 0.58]
Benrazavy, 2019	245	417	0.59	[0.54; 0.64]
Bloomgarden, 2017	10674	18560	0.58	[0.57; 0.58]
Bonger, 2018	141	151	0.93	[0.88; 0.97]
Bruce, 2015	77	200	0.38	[0.32; 0.46]
Bryson, 2013	313759	444418	0.71	[0.70; 0.71]
Buysman, 2017	1215	2261	0.54	[0.52; 0.56]
Cai, 2016	1471	2925	0.50	[0.48; 0.52]
Cai, 2017	8298	17429	0.48	[0.47; 0.48]
Carls, 2017a	242	652	0.37	[0.33; 0.41]
Carls, 2017b	1878	4985	0.38	[0.36; 0.39]
Chang, 2015	184035	559864	0.33	[0.33; 0.33]
Chen, 2013	6307	11299	0.56	[0.55; 0.57]
Chen, 2016	6398	12123	0.53	[0.52; 0.54]
Cheng, 2013	6739	11580	0.58	[0.57; 0.59]
Chepulis, 2020	1207	1595	0.76	[0.73; 0.78]
Chew, 2015	349	605	0.58	[0.54; 0.62]
Chong, 2014	42756	75555	0.57	[0.56; 0.57]
de Vries, 2014	53	85	0.62	[0.51; 0.73]
Degefa, 2020	116	116	1.00	[0.97; 1.00]
Dossa, 2015	78321	100216	0.78	[0.78; 0.78]
Elsous, 2017	136	241	0.56	[0.50; 0.63]
Fai, 2017	23	115	0.20	[0.13; 0.28]
Farmer, 2016	35529	40722	0.87	[0.87; 0.88]
Farr, 2014	100092	238372	0.42	[0.42; 0.42]
Farr, 2016	12362	27385	0.45	[0.45; 0.46]
Garcia, 2019	13	279	0.05	[0.03; 0.08]
Gatwood, 2018a	99430	159032	0.63	[0.62; 0.63]
Gatwood, 2018b	93928	148544	0.63	[0.63; 0.63]
Gatwood, 2021	58	433	0.13	[0.10; 0.17]
Gordon, 2018	27547	33849	0.81	[0.81; 0.82]
Graetz, 2020	79750	111463	0.72	[0.71; 0.72]
Guenette, 2013	93418	119832	0.78	[0.78; 0.78]
Guenette, 2015	18	45	0.40	[0.26; 0.56]
Guenette, 2016	261	598	0.44	[0.40; 0.48]
Hagen, 2014	2820	4978	0.57	[0.55; 0.58]
Haines, 2018	77	126	0.61	[0.52; 0.70]
Hansen, 2015	1328	1848	0.72	[0.70; 0.74]
Hayashi, 2018	748	754	0.99	[0.98; 1.00]

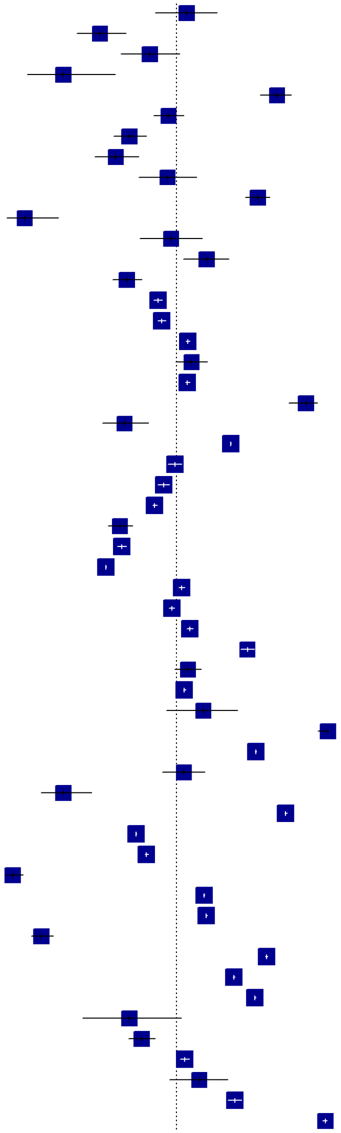

Hedna, 2013	123	205	0.60	[0.53; 0.67]
Heissam, 2015	98	372	0.26	[0.22; 0.31]
Hong, 2014	10550	23034	0.46	[0.45; 0.46]
Horii, 2019	440	884	0.50	[0.46; 0.53]
Horsburgh, 2019	53592	85066	0.63	[0.63; 0.63]
Horvat, 2018	128	272	0.47	[0.41; 0.53]
Huber, 2016	1854	11035	0.17	[0.16; 0.18]
Ibrahim, 2021	167	304	0.55	[0.49; 0.61]
Iyengar, 2014	82726	150493	0.55	[0.55; 0.55]
Iyengar, 2016	26805	40703	0.66	[0.65; 0.66]
Jamous, 2011	50	131	0.38	[0.30; 0.47]
Jannuzzi, 2014	12	17	0.71	[0.44; 0.90]
Jannuzzi, 2020	115	157	0.73	[0.66; 0.80]
Jarab, 2014	17	37	0.46	[0.29; 0.63]
Jiang, 2020	34242	58349	0.59	[0.58; 0.59]
Khan, 2014	18	184	0.10	[0.06; 0.15]
Kharjul, 2019	3246	7138	0.45	[0.44; 0.47]
Kim, 2016	78	304	0.26	[0.21; 0.31]
Kim, 2018	22261	65067	0.34	[0.34; 0.35]
Koyanagi, 2016	320	452	0.71	[0.66; 0.75]
Kumar, 2019	554	1000	0.55	[0.52; 0.59]
Kurtyka, 2016	4181	5890	0.71	[0.70; 0.72]
Lee, 2013	84	278	0.30	[0.25; 0.36]
Lee, 2017	164	382	0.43	[0.38; 0.48]
Leporini, 2016	199	1133	0.18	[0.15; 0.20]
Lo-Ciganic, 2015	12589	33130	0.38	[0.37; 0.39]
Lo-Ciganic, 2016	6558	16256	0.40	[0.40; 0.41]
Lokhandwala, 2016	12637	23361	0.54	[0.53; 0.55]
Lopez-Simarro, 2016	158	252	0.63	[0.56; 0.69]
Mallah, 2019	101	300	0.34	[0.28; 0.39]
Manteuffel, 2014	3391734	5141047	0.66	[0.66; 0.66]
Martinez-Perez, 2021	97	187	0.52	[0.44; 0.59]
Mhatre, 2016	4189	7028	0.60	[0.58; 0.61]
Miller, 2015	695	1998	0.35	[0.33; 0.37]
Min, 2019	28005	49424	0.57	[0.56; 0.57]
Mirahmadizadeh, 2020	44	358	0.12	[0.09; 0.16]
Mroueh, 2018	78	245	0.32	[0.26; 0.38]
Mukherjee, 2013	156	225	0.69	[0.63; 0.75]
Naffaa, 2020	35085	113749	0.31	[0.31; 0.31]
Nichols, 2016	1890	2844	0.66	[0.65; 0.68]
Nichols, 2018	45218	54480	0.83	[0.83; 0.83]
Nigam, 2012	4241	7306	0.58	[0.57; 0.59]
Nishimura, 2019a	46959	50349	0.93	[0.93; 0.93]
Nishimura, 2019b	6691	7524	0.89	[0.88; 0.90]
Ofori-Asenso, 2019a	10039	15915	0.63	[0.62; 0.64]
Ofori-Asenso, 2019b	7713	11981	0.64	[0.64; 0.65]
Oh, 2020	36250	37248	0.97	[0.97; 0.97]
Olickal, 2021	299	515	0.58	[0.54; 0.62]
O'Shea, 2013	10644	15958	0.67	[0.66; 0.67]
O'Shea, 2015	7134	11035	0.65	[0.64; 0.66]
Oung, 2017	174	387	0.45	[0.40; 0.50]
Pascal, 2012	87	120	0.72	[0.64; 0.80]
Peeters, 2015	15	21	0.71	[0.48; 0.89]
Quilliam, 2013	54075	73822	0.73	[0.73; 0.74]

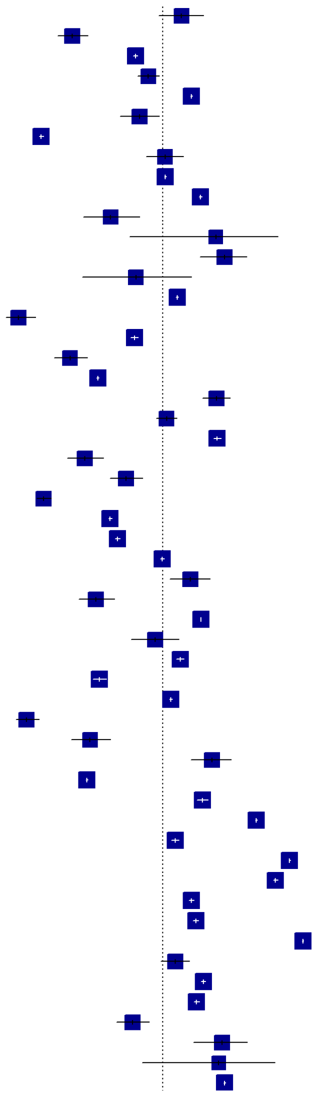

Rahmatullah, 2021	66	198	0.33	[0.27; 0.40]
Rana, 2017	54	145	0.37	[0.29; 0.46]
Ranashinge, 2018	93	212	0.44	[0.37; 0.51]
Rascati, 2017	13040	26089	0.50	[0.49; 0.51]
Rathish, 2019	46	200	0.23	[0.17; 0.29]
Reach, 2011	81	90	0.90	[0.82; 0.95]
Reach, 2018	489	1084	0.45	[0.42; 0.48]
Rinne, 2015	29127	40766	0.71	[0.71; 0.72]
Romagnoli, 2021	9899	29952	0.33	[0.33; 0.34]
Sacks, 2013	32218	49642	0.65	[0.64; 0.65]
Sacks, 2015	6130	12771	0.48	[0.47; 0.49]
Sankar, 2015	75	317	0.24	[0.19; 0.29]
Saraiva, 2020	54	250	0.22	[0.17; 0.27]
Satirapoj, 2020	315	659	0.48	[0.44; 0.52]
Sattler, 2014	84	243	0.35	[0.29; 0.41]
Saundankar, 2016	144216	238402	0.60	[0.60; 0.61]
Schwab, 2019	29733	38614	0.77	[0.77; 0.77]
Shields, 2020	19664	24400	0.81	[0.80; 0.81]
Shrestha, 2013	54	90	0.60	[0.49; 0.70]
Sicras-Mainar, 2014	507	987	0.51	[0.48; 0.55]
Simard, 2015	107355	160231	0.67	[0.67; 0.67]
Simard, 2018	34890	52188	0.67	[0.66; 0.67]
Simon-Tuval, 2018	158	308	0.51	[0.46; 0.57]
Singhal, 2019	356	750	0.47	[0.44; 0.51]
Taira, 2017	3819	5163	0.74	[0.73; 0.75]
Tanabe, 2017	4420	7108	0.62	[0.61; 0.63]
Tandon, 2015	17	66	0.26	[0.16; 0.38]
Tang, 2020	48922	111186	0.44	[0.44; 0.44]
Tiv, 2012	1158	2859	0.41	[0.39; 0.42]
Tunceli, 2015a	78764	133499	0.59	[0.59; 0.59]
Tunceli, 2015b	3772	6276	0.60	[0.59; 0.61]
Vichayanrat, 2013	832	1064	0.78	[0.76; 0.81]
Vietri, 2016	779	2030	0.38	[0.36; 0.41]
Vittorino Gaddi, 2014	73828	169375	0.44	[0.43; 0.44]
Vlacho, 2021	36118	75808	0.48	[0.47; 0.48]
Waari, 2018	67	129	0.52	[0.43; 0.61]
Walz, 2014	271	430	0.63	[0.58; 0.68]
White, 2012	44	60	0.73	[0.60; 0.84]
Wong, 2011	23997	26782	0.90	[0.89; 0.90]
Wong, 2012	313759	444418	0.71	[0.70; 0.71]
Wong, 2014	14068	26051	0.54	[0.53; 0.55]
Wong, 2015	383	565	0.68	[0.64; 0.72]
Wu, 2016	59	130	0.45	[0.37; 0.54]
Zhou, 2013	39	87	0.45	[0.34; 0.56]
Zhu, 2015	2269	24067	0.09	[0.09; 0.10]
Zomahoun, 2016	293	340	0.86	[0.82; 0.90]
Zongo, 2016	66	153	0.43	[0.35; 0.51]
Total		10041928	0.54	[0.51; 0.58]

Heterogeneity: χ^2_{155} = 673989.71 (P < .001), I^2 = 100%

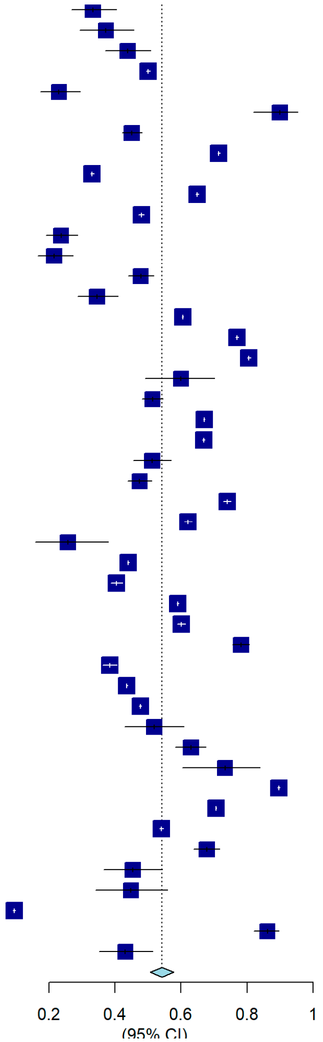

Figure S2. Forest plot of proportions, and relative confidence intervals, according with risk of bias.

Source	Events	Total	Proportion	95% CI
RoB = Low				
Abdullah, 2019	67	117	0.57	[0.48; 0.66]
Aloudah, 2018	158	395	0.40	[0.35; 0.45]
AlQarni, 2019	76	212	0.36	[0.29; 0.43]
Arulmozhi, 2014	61	116	0.53	[0.43; 0.62]
Ashur, 2015	126	199	0.63	[0.56; 0.70]
Ayoub, 2019	196	500	0.39	[0.35; 0.44]
Balkhi, 2019	2652	5457	0.49	[0.47; 0.50]
Bell, 2017	15746	27314	0.58	[0.57; 0.58]
Bloomgarden, 2017	10674	18560	0.58	[0.57; 0.58]
Cai, 2016	1471	2925	0.50	[0.48; 0.52]
Cai, 2017	8298	17429	0.48	[0.47; 0.48]
Cheng, 2013	6739	11580	0.58	[0.57; 0.59]
Chepulis, 2020	1207	1595	0.76	[0.73; 0.78]
Elsous, 2017	136	241	0.56	[0.50; 0.63]
Farmer, 2016	35529	40722	0.87	[0.87; 0.88]
Farr, 2014	100092	238372	0.42	[0.42; 0.42]
Farr, 2016	12362	27385	0.45	[0.45; 0.46]
Garcia, 2019	13	279	0.05	[0.03; 0.08]
Gatwood, 2021	58	433	0.13	[0.10; 0.17]
Graetz, 2020	79750	111463	0.72	[0.71; 0.72]
Guenette, 2016	261	598	0.44	[0.40; 0.48]
Hedna, 2013	123	205	0.60	[0.53; 0.67]
Heissam, 2015	98	372	0.26	[0.22; 0.31]
Horsburgh, 2019	53592	85066	0.63	[0.63; 0.63]
Huber, 2016	1854	11035	0.17	[0.16; 0.18]
Ibrahim, 2021	167	304	0.55	[0.49; 0.61]
Iyengar, 2014	82726	150493	0.55	[0.55; 0.55]
Iyengar, 2016	26805	40703	0.66	[0.65; 0.66]
Khan, 2014	18	184	0.10	[0.06; 0.15]
Kharjul, 2019	3246	7138	0.45	[0.44; 0.47]
Kim, 2016	78	304	0.26	[0.21; 0.31]
Koyanagi, 2016	320	452	0.71	[0.66; 0.75]
Lee, 2013	84	278	0.30	[0.25; 0.36]
Lee, 2017	164	382	0.43	[0.38; 0.48]
Mallah, 2019	101	300	0.34	[0.28; 0.39]
Manteuffel, 2014	3391734	5141047	0.66	[0.66; 0.66]
Martinez-Perez, 2021	97	187	0.52	[0.44; 0.59]
Miller, 2015	695	1998	0.35	[0.33; 0.37]
Mirahmadizadeh, 2020	44	358	0.12	[0.09; 0.16]
Mroueh, 2018	78	245	0.32	[0.26; 0.38]
Naffaa, 2020	35085	113749	0.31	[0.31; 0.31]
Nigam, 2012	4241	7306	0.58	[0.57; 0.59]
Nishimura, 2019b	6691	7524	0.89	[0.88; 0.90]
Ofori-Asenso, 2019a	10039	15915	0.63	[0.62; 0.64]
Ofori-Asenso, 2019b	7713	11981	0.64	[0.64; 0.65]
Oh, 2020	36250	37248	0.97	[0.97; 0.97]
O'Shea, 2013	10644	15958	0.67	[0.66; 0.67]
O'Shea, 2015	7134	11035	0.65	[0.64; 0.66]
Oung, 2017	174	387	0.45	[0.40; 0.50]
Quilliam, 2013	54075	73822	0.73	[0.73; 0.74]
Ranashinge, 2018	93	212	0.44	[0.37; 0.51]
Rascati, 2017	13040	26089	0.50	[0.49; 0.51]
Rathish, 2019	46	200	0.23	[0.17; 0.29]
Romagnoli, 2021	9899	29952	0.33	[0.33; 0.34]
Sankar, 2015	75	317	0.24	[0.19; 0.29]
Satirapoj, 2020	315	659	0.48	[0.44; 0.52]
Saundankar, 2016	144216	238402	0.60	[0.60; 0.61]
Shields, 2020	19664	24400	0.81	[0.80; 0.81]
Simard, 2015	107355	160231	0.67	[0.67; 0.67]
Singhal, 2019	356	750	0.47	[0.44; 0.51]
Taira, 2017	3819	5163	0.74	[0.73; 0.75]
Tang, 2020	48922	111186	0.44	[0.44; 0.44]
Tunceli, 2015a	78764	133499	0.59	[0.59; 0.59]
Tunceli, 2015b	3772	6276	0.60	[0.59; 0.61]
Vietri, 2016	779	2030	0.38	[0.36; 0.41]
Wong, 2011	23997	26782	0.90	[0.89; 0.90]
Wong, 2014	14068	26051	0.54	[0.53; 0.55]
Zhu, 2015	2269	24067	0.09	[0.09; 0.10]
Zongo, 2016	66	153	0.43	[0.35; 0.51]
Total		7058287	0.50	[0.45; 0.55]

Heterogeneity: χ^2_{68} = 280786.97 (P < .001), I^2 = 100%

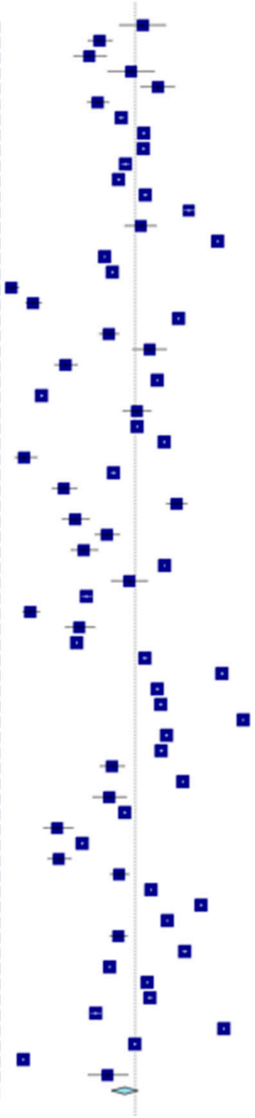

RoB = Moderate				
Acharya, 2019	50	161	0.31	[0.24; 0.39]
Adisa, 2013	60	130	0.46	[0.37; 0.55]
Afaya, 2020	209	247	0.85	[0.80; 0.89]
Ahmad, 2013	245	473	0.52	[0.47; 0.56]
Aminde, 2019	69	134	0.51	[0.43; 0.60]
An, 2014	388	492	0.79	[0.75; 0.82]
Andanalusia, 2019	5	60	0.08	[0.03; 0.18]
Basak, 2014	3006	6043	0.50	[0.48; 0.51]
Benrazavy, 2019	245	417	0.59	[0.54; 0.64]
Bonger, 2018	141	151	0.93	[0.88; 0.97]
Bruce, 2015	77	200	0.38	[0.32; 0.46]
Buysman, 2017	1215	2261	0.54	[0.52; 0.56]
Carls, 2017a	242	652	0.37	[0.33; 0.41]
Carls, 2017b	1878	4985	0.38	[0.36; 0.39]
Chang, 2015	184035	559864	0.33	[0.33; 0.33]
Chen, 2016	6398	12123	0.53	[0.52; 0.54]
Chew, 2015	349	605	0.58	[0.54; 0.62]
Chong, 2014	42756	75555	0.57	[0.56; 0.57]
de Vries, 2014	53	85	0.62	[0.51; 0.73]
Dossa, 2015	78321	100216	0.78	[0.78; 0.78]
Gatwood, 2018a	99430	159032	0.63	[0.62; 0.63]
Gatwood, 2018b	93928	148544	0.63	[0.63; 0.63]
Gordon, 2018	27547	33849	0.81	[0.81; 0.82]
Guenette, 2013	93418	119832	0.78	[0.78; 0.78]
Guenette, 2015	18	45	0.40	[0.26; 0.56]
Hagen, 2014	2820	4978	0.57	[0.55; 0.58]
Haines, 2018	77	126	0.61	[0.52; 0.70]
Hansen, 2015	1328	1848	0.72	[0.70; 0.74]
Hong, 2014	10550	23034	0.46	[0.45; 0.46]
Horii, 2019	440	884	0.50	[0.46; 0.53]
Jannuzzi, 2014	12	17	0.71	[0.44; 0.90]
Jannuzzi, 2020	115	157	0.73	[0.66; 0.80]
Jarab, 2014	17	37	0.46	[0.29; 0.63]
Jiang, 2020	34242	58349	0.59	[0.58; 0.59]
Kim, 2018	22261	65067	0.34	[0.34; 0.35]
Kurtyka, 2016	4181	5890	0.71	[0.70; 0.72]
Leporini, 2016	199	1133	0.18	[0.15; 0.20]
Lo-Ciganic, 2015	12589	33130	0.38	[0.37; 0.39]
Lo-Ciganic, 2016	6558	16256	0.40	[0.40; 0.41]
Lokhandwala, 2016	12637	23361	0.54	[0.53; 0.55]
Lopez-Simarro, 2016	158	252	0.63	[0.56; 0.69]
Mhatre, 2016	4189	7028	0.60	[0.58; 0.61]
Min, 2019	28005	49424	0.57	[0.56; 0.57]
Mukherjee, 2013	156	225	0.69	[0.63; 0.75]
Nichols, 2016	1890	2844	0.66	[0.65; 0.68]
Nichols, 2018	45218	54480	0.83	[0.83; 0.83]
Nishimura, 2019a	46959	50349	0.93	[0.93; 0.93]
Olickal, 2021	299	515	0.58	[0.54; 0.62]
Rahmatullah, 2021	66	198	0.33	[0.27; 0.40]
Reach, 2018	489	1084	0.45	[0.42; 0.48]
Sacks, 2013	32218	49642	0.65	[0.64; 0.65]
Sacks, 2015	6130	12771	0.48	[0.47; 0.49]
Saraiva, 2020	54	250	0.22	[0.17; 0.27]
Sattler, 2014	84	243	0.35	[0.29; 0.41]
Schwab, 2019	29733	38614	0.77	[0.77; 0.77]
Sicras-Mainar, 2014	507	987	0.51	[0.48; 0.55]
Simard, 2018	34890	52188	0.67	[0.66; 0.67]
Simon-Tuval, 2018	158	308	0.51	[0.46; 0.57]
Tanabe, 2017	4420	7108	0.62	[0.61; 0.63]
Tandon, 2015	17	66	0.26	[0.16; 0.38]
Tiv, 2012	1158	2859	0.41	[0.39; 0.42]
Vichayanrat, 2013	832	1064	0.78	[0.76; 0.81]
Vittorino Gaddi, 2014	73828	169375	0.44	[0.43; 0.44]
Vlacho, 2021	36118	75808	0.48	[0.47; 0.48]
Waari, 2018	67	129	0.52	[0.43; 0.61]
Walz, 2014	271	430	0.63	[0.58; 0.68]
White, 2012	44	60	0.73	[0.60; 0.84]
Wong, 2012	313759	444418	0.71	[0.70; 0.71]
Wong, 2015	383	565	0.68	[0.64; 0.72]
Wu, 2016	59	130	0.45	[0.37; 0.54]
Zomahoun, 2016	293	340	0.86	[0.82; 0.90]
Total		2484177	0.57	[0.52; 0.61]

Heterogeneity: χ^2_{70} = 342012.48 (P < .001), I^2 = 100%

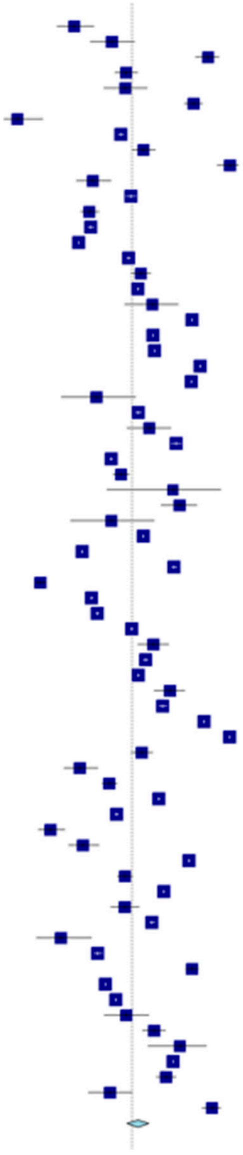

**RoB = High**				
Aditama, 2019	8	40	0.20	[0.09; 0.36]
Bryson, 2013	313759	444418	0.71	[0.70; 0.71]
Chen, 2013	6307	11299	0.56	[0.55; 0.57]
Degefa, 2020	116	116	1.00	[0.97; 1.00]
Fai, 2017	23	115	0.20	[0.13; 0.28]
Hayashi, 2018	748	754	0.99	[0.98; 1.00]
Horvat, 2018	128	272	0.47	[0.41; 0.53]
Jamous, 2011	50	131	0.38	[0.30; 0.47]
Kumar, 2019	554	1000	0.55	[0.52; 0.59]
Pascal, 2012	87	120	0.72	[0.64; 0.80]
Peeters, 2015	15	21	0.71	[0.48; 0.89]
Rana, 2017	54	145	0.37	[0.29; 0.46]
Reach, 2011	81	90	0.90	[0.82; 0.95]
Rinne, 2015	29127	40766	0.71	[0.71; 0.72]
Shrestha, 2013	54	90	0.60	[0.49; 0.70]
Zhou, 2013	39	87	0.45	[0.34; 0.56]
Total		499464	0.63	[0.48; 0.77]
Heterogeneity: $\chi^2_{15} = 2411.59$ ($P < .001$), $I^2 = 99\%$				
Total		10041928	0.54	[0.51; 0.58]
Heterogeneity: $\chi^2_{155} = 673989.71$ ($P < .001$), $I^2 = 100\%$				
Test for subgroup differences: $\chi^2_2 = 4.67$ ($P = .10$)				

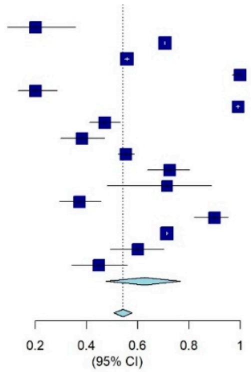

Figure S3. Forest plot of proportions, and relative confidence intervals, of adherent patients stratified for gender.

Source	Events	Total	Proportion	95% CI
gender = Men				
Aloudah, 2018	105	236	0.44	[0.38; 0.51]
AlQarni, 2019	54	142	0.38	[0.30; 0.47]
Balkhi, 2019	1066	2057	0.52	[0.50; 0.54]
Buysman, 2017	771	1344	0.57	[0.55; 0.60]
Chepulis, 2020	675	886	0.76	[0.73; 0.79]
Garcia, 2019	4	104	0.04	[0.01; 0.10]
Gatwood, 2018a	95493	152055	0.63	[0.63; 0.63]
Gatwood, 2018b	90212	142054	0.64	[0.63; 0.64]
Guenette, 2013	57960	72980	0.79	[0.79; 0.80]
Hagen, 2014	2532	4242	0.60	[0.58; 0.61]
Hansen, 2015	1277	1770	0.72	[0.70; 0.74]
Horii, 2019b	42	87	0.48	[0.37; 0.59]
Horvat, 2018	68	136	0.50	[0.41; 0.59]
Jamous, 2011	27	83	0.33	[0.23; 0.44]
Jiang, 2020	15859	26784	0.59	[0.59; 0.60]
Khan, 2014	10	81	0.12	[0.06; 0.22]
Kharjul, 2019	1729	3832	0.45	[0.44; 0.47]
Kim, 2018b	13227	39808	0.33	[0.33; 0.34]
Kurtyka, 2016	3114	4397	0.71	[0.69; 0.72]
Lee, 2017	83	178	0.47	[0.39; 0.54]
Lo-Ciganic, 2016	2482	5234	0.47	[0.46; 0.49]
Manteuffel, 2014	1643695	2435104	0.67	[0.67; 0.68]
Min, 2019	26997	47367	0.57	[0.57; 0.57]
Naffaa, 2020	18670	59193	0.32	[0.31; 0.32]
O'Shea, 2013	5960	8601	0.69	[0.68; 0.70]
Quilliam, 2013	28043	37713	0.74	[0.74; 0.75]
Rana, 2017	14	54	0.26	[0.15; 0.40]
Sattler, 2014	17	55	0.31	[0.19; 0.45]
Saundankar, 2016	66144	108558	0.61	[0.61; 0.61]
Tunceli, 2015b	2134	3580	0.60	[0.58; 0.61]
White, 2012	26	40	0.65	[0.48; 0.79]
Wong, 2011	10611	12060	0.88	[0.87; 0.89]
Wong, 2014	14311	25490	0.56	[0.56; 0.57]
Wong, 2015	180	263	0.68	[0.62; 0.74]
Zhu, 2015	1155	11584	0.10	[0.09; 0.11]
Zomahoun, 2016	171	196	0.87	[0.82; 0.92]
Total		3208348	0.53	[0.46; 0.60]

Heterogeneity: $\chi^2_{35} = 87139.84$ ($P < .001$), $I^2 = 100\%$

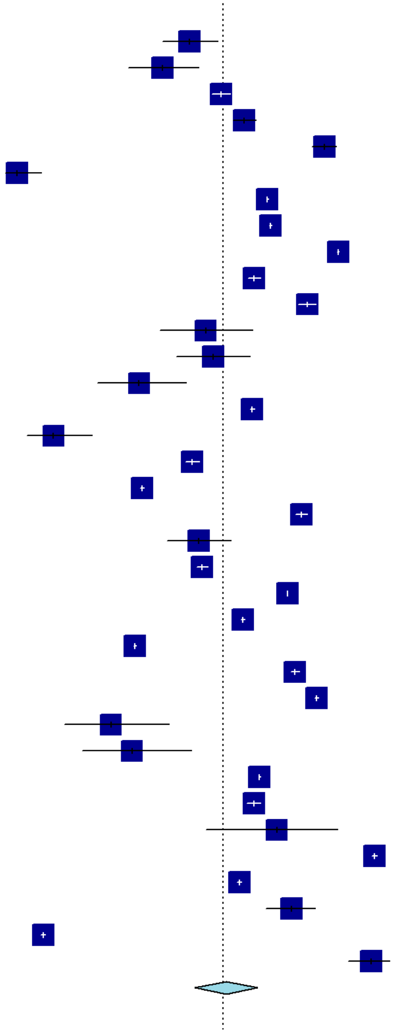

gender = Women

Aloudah, 2018	53	159	0.33	[0.26; 0.41]
AlQarni, 2019	22	70	0.31	[0.21; 0.44]
Balkhi, 2019	1586	3400	0.47	[0.45; 0.48]
Buysman, 2017	444	917	0.48	[0.45; 0.52]
Chepulis, 2020	532	709	0.75	[0.72; 0.78]
Garcia, 2019	9	175	0.05	[0.02; 0.10]
Gatwood, 2018a	3937	6977	0.56	[0.55; 0.58]
Gatwood, 2018b	3716	6490	0.57	[0.56; 0.58]
Guenette, 2013	61872	78193	0.79	[0.79; 0.79]
Hagen, 2014	288	736	0.39	[0.36; 0.43]
Hansen, 2015	51	78	0.65	[0.54; 0.76]
Horii, 2019b	495	797	0.62	[0.59; 0.65]
Horvat, 2018	101	187	0.54	[0.47; 0.61]
Jamous, 2011	23	47	0.49	[0.34; 0.64]
Jiang, 2020	18383	31565	0.58	[0.58; 0.59]
Khan, 2014	8	103	0.08	[0.03; 0.15]
Kharjul, 2019	1517	3305	0.46	[0.44; 0.48]
Kim, 2018b	9034	25259	0.36	[0.35; 0.36]
Kurtyka, 2016	1068	1493	0.72	[0.69; 0.74]
Lee, 2017	81	204	0.40	[0.33; 0.47]
Lo-Ciganic, 2016	4076	11022	0.37	[0.36; 0.38]
Manteuffel, 2014	1748039	2705943	0.65	[0.65; 0.65]
Min, 2019	1008	2057	0.49	[0.47; 0.51]
Naffaa, 2020	16415	54556	0.30	[0.30; 0.30]
O'Shea, 2013	4679	7357	0.64	[0.62; 0.65]
Quilliam, 2013	26032	36109	0.72	[0.72; 0.73]
Rana, 2017	40	91	0.44	[0.34; 0.55]
Sattler, 2014	67	188	0.36	[0.29; 0.43]
Saundankar, 2016	78072	129844	0.60	[0.60; 0.60]
Tunceli, 2015b	1639	2696	0.61	[0.59; 0.63]
White, 2012	18	20	0.90	[0.68; 0.99]
Wong, 2011	13396	14722	0.91	[0.91; 0.91]
Wong, 2014	257	561	0.46	[0.42; 0.50]
Wong, 2015	203	302	0.67	[0.62; 0.72]
Zhu, 2015	1114	12483	0.09	[0.08; 0.09]
Zomahoun, 2016	122	144	0.85	[0.78; 0.90]
Total		3138959	0.52	[0.44; 0.59]
Heterogeneity: χ^2_{35} = 78180.72 (P < .001), I^2 = 100%				
Total		6347307	0.52	[0.47; 0.58]
Heterogeneity: χ^2_{71} = 167998.13 (P < .001), I^2 = 100%				
Test for subgroup differences: χ^2_1 = 0.07 (P = .78)				

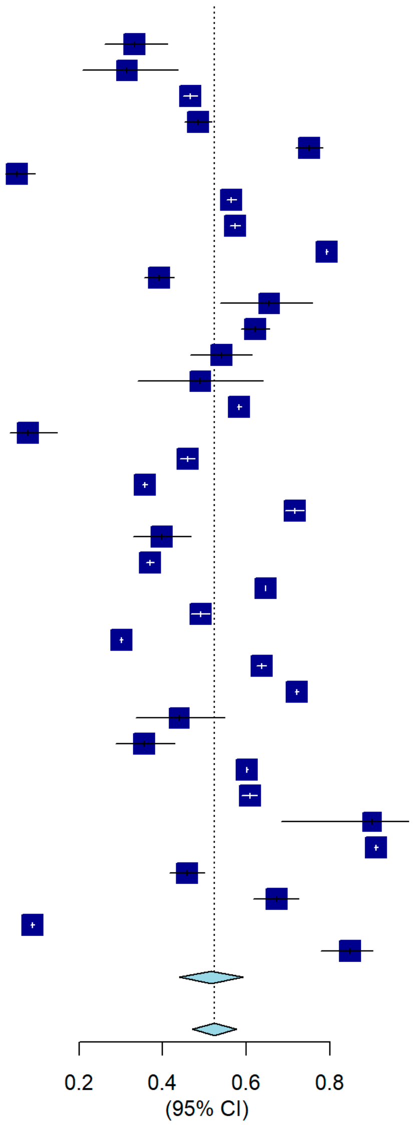

Figure S4. Forest plot of proportions, and relative confidence intervals, of adherent patients stratified for age category.

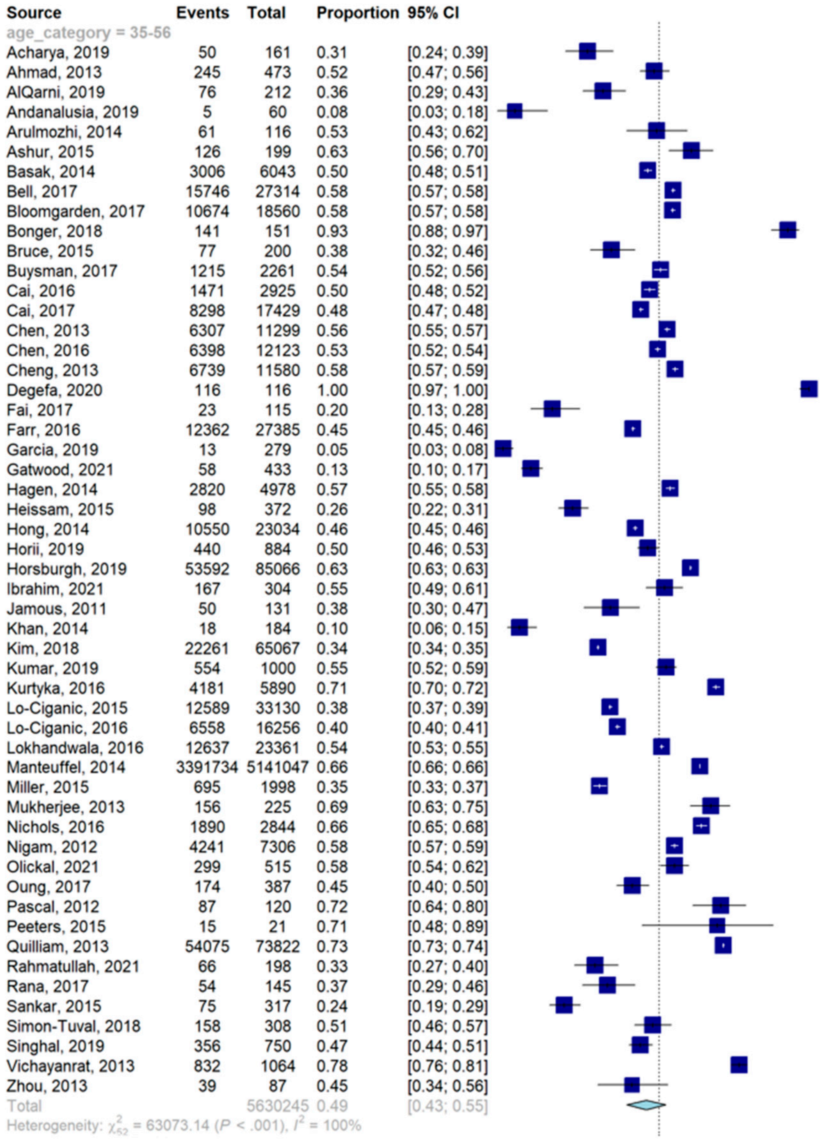

Source	Events	Total	Proportion	95% CI
age_category = 35-56				
Acharya, 2019	50	161	0.31	[0.24; 0.39]
Ahmad, 2013	245	473	0.52	[0.47; 0.56]
AlQarni, 2019	76	212	0.36	[0.29; 0.43]
Andanalusia, 2019	5	60	0.08	[0.03; 0.18]
Arulmozhi, 2014	61	116	0.53	[0.43; 0.62]
Ashur, 2015	126	199	0.63	[0.56; 0.70]
Basak, 2014	3006	6043	0.50	[0.48; 0.51]
Bell, 2017	15746	27314	0.58	[0.57; 0.58]
Bloomgarden, 2017	10674	18560	0.58	[0.57; 0.58]
Bonger, 2018	141	151	0.93	[0.88; 0.97]
Bruce, 2015	77	200	0.38	[0.32; 0.46]
Buysman, 2017	1215	2261	0.54	[0.52; 0.56]
Cai, 2016	1471	2925	0.50	[0.48; 0.52]
Cai, 2017	8298	17429	0.48	[0.47; 0.48]
Chen, 2013	6307	11299	0.56	[0.55; 0.57]
Chen, 2016	6398	12123	0.53	[0.52; 0.54]
Cheng, 2013	6739	11580	0.58	[0.57; 0.59]
Degefa, 2020	116	116	1.00	[0.97; 1.00]
Fai, 2017	23	115	0.20	[0.13; 0.28]
Farr, 2016	12362	27385	0.45	[0.45; 0.46]
Garcia, 2019	13	279	0.05	[0.03; 0.08]
Gatwood, 2021	58	433	0.13	[0.10; 0.17]
Hagen, 2014	2820	4978	0.57	[0.55; 0.58]
Heissam, 2015	98	372	0.26	[0.22; 0.31]
Hong, 2014	10550	23034	0.46	[0.45; 0.46]
Horii, 2019	440	884	0.50	[0.46; 0.53]
Horsburgh, 2019	53592	85066	0.63	[0.63; 0.63]
Ibrahim, 2021	167	304	0.55	[0.49; 0.61]
Jamous, 2011	50	131	0.38	[0.30; 0.47]
Khan, 2014	18	184	0.10	[0.06; 0.15]
Kim, 2018	22261	65067	0.34	[0.34; 0.35]
Kumar, 2019	554	1000	0.55	[0.52; 0.59]
Kurtyka, 2016	4181	5890	0.71	[0.70; 0.72]
Lo-Ciganic, 2015	12589	33130	0.38	[0.37; 0.39]
Lo-Ciganic, 2016	6558	16256	0.40	[0.40; 0.41]
Lokhandwala, 2016	12637	23361	0.54	[0.53; 0.55]
Manteuffel, 2014	3391734	5141047	0.66	[0.66; 0.66]
Miller, 2015	695	1998	0.35	[0.33; 0.37]
Mukherjee, 2013	156	225	0.69	[0.63; 0.75]
Nichols, 2016	1890	2844	0.66	[0.65; 0.68]
Nigam, 2012	4241	7306	0.58	[0.57; 0.59]
Olickal, 2021	299	515	0.58	[0.54; 0.62]
Oung, 2017	174	387	0.45	[0.40; 0.50]
Pascal, 2012	87	120	0.72	[0.64; 0.80]
Peeters, 2015	15	21	0.71	[0.48; 0.89]
Quilliam, 2013	54075	73822	0.73	[0.73; 0.74]
Rahmatullah, 2021	66	198	0.33	[0.27; 0.40]
Rana, 2017	54	145	0.37	[0.29; 0.46]
Sankar, 2015	75	317	0.24	[0.19; 0.29]
Simon-Tuval, 2018	158	308	0.51	[0.46; 0.57]
Singhal, 2019	356	750	0.47	[0.44; 0.51]
Vichayanrat, 2013	832	1064	0.78	[0.76; 0.81]
Zhou, 2013	39	87	0.45	[0.34; 0.56]
Total		5630245	0.49	[0.43; 0.55]

Heterogeneity: $\chi^2_{52} = 63073.14$ ($P < .001$), $I^2 = 100\%$

age_category = 56-63				
Abdullah, 2019	67	117	0.57	[0.48; 0.66]
Adisa, 2013	60	130	0.46	[0.37; 0.55]
Afaya, 2020	209	247	0.85	[0.80; 0.89]
Aloudah, 2018	158	395	0.40	[0.35; 0.45]
Aminde, 2019	69	134	0.51	[0.43; 0.60]
An, 2014	388	492	0.79	[0.75; 0.82]
Ayoub, 2019	196	500	0.39	[0.35; 0.44]
Balkhi, 2019	2652	5457	0.49	[0.47; 0.50]
Benrazavy, 2019	245	417	0.59	[0.54; 0.64]
Carls, 2017a	242	652	0.37	[0.33; 0.41]
Carls, 2017b	1878	4985	0.38	[0.36; 0.39]
Chang, 2015	184035	559864	0.33	[0.33; 0.33]
Chew, 2015	349	605	0.58	[0.54; 0.62]
Chong, 2014	42756	75555	0.57	[0.56; 0.57]
Elsous, 2017	136	241	0.56	[0.50; 0.63]
Farr, 2014	100092	238372	0.42	[0.42; 0.42]
Gatwood, 2018a	99430	159032	0.63	[0.62; 0.63]
Gatwood, 2018b	93928	148544	0.63	[0.63; 0.63]
Gordon, 2018	27547	33849	0.81	[0.81; 0.82]
Guenette, 2016	261	598	0.44	[0.40; 0.48]
Haines, 2018	77	126	0.61	[0.52; 0.70]
Jannuzzi, 2014	12	17	0.71	[0.44; 0.90]
Jannuzzi, 2020	115	157	0.73	[0.66; 0.80]
Kharjul, 2019	3246	7138	0.45	[0.44; 0.47]
Kim, 2016	78	304	0.26	[0.21; 0.31]
Lee, 2013	84	278	0.30	[0.25; 0.36]
Lee, 2017	164	382	0.43	[0.38; 0.48]
Mallah, 2019	101	300	0.34	[0.28; 0.39]
Min, 2019	28005	49424	0.57	[0.56; 0.57]
Mirahmadizadeh, 2020	44	358	0.12	[0.09; 0.16]
Mroueh, 2018	78	245	0.32	[0.26; 0.38]
Naffaa, 2020	35085	113749	0.31	[0.31; 0.31]
Nishimura, 2019a	46959	50349	0.93	[0.93; 0.93]
Nishimura, 2019b	6691	7524	0.89	[0.88; 0.90]
Ofori-Asenso, 2019a	10039	15915	0.63	[0.62; 0.64]
Ofori-Asenso, 2019b	7713	11981	0.64	[0.64; 0.65]
O'Shea, 2013	10644	15958	0.67	[0.66; 0.67]
Ranashinge, 2018	93	212	0.44	[0.37; 0.51]
Rascati, 2017	13040	26089	0.50	[0.49; 0.51]
Rathish, 2019	46	200	0.23	[0.17; 0.29]
Reach, 2011	81	90	0.90	[0.82; 0.95]
Saraiva, 2020	54	250	0.22	[0.17; 0.27]
Shrestha, 2013	54	90	0.60	[0.49; 0.70]
Tandon, 2015	17	66	0.26	[0.16; 0.38]
Tunceli, 2015a	78764	133499	0.59	[0.59; 0.59]
Vietri, 2016	779	2030	0.38	[0.36; 0.41]
Vlacho, 2021	36118	75808	0.48	[0.47; 0.48]
Waari, 2018	67	129	0.52	[0.43; 0.61]
Wong, 2015	383	565	0.68	[0.64; 0.72]
Wu, 2016	59	130	0.45	[0.37; 0.54]
Zomahoun, 2016	293	340	0.86	[0.82; 0.90]
Total		1743889	0.53	[0.47; 0.59]

Heterogeneity: χ^2_{50} = 199691.23 (P < .001), I^2 = 100%

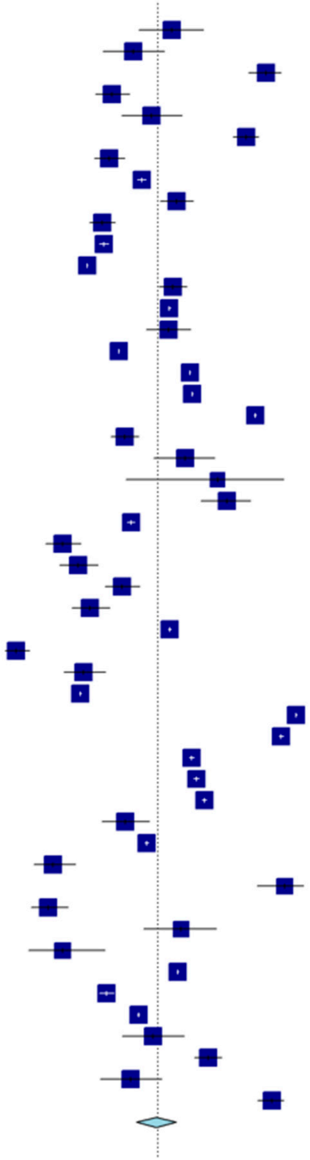

age_category = 63-77

Aditama, 2019	8	40	0.20	[0.09; 0.36]
Bryson, 2013	313759	444418	0.71	[0.70; 0.71]
Chepulis, 2020	1207	1595	0.76	[0.73; 0.78]
de Vries, 2014	53	85	0.62	[0.51; 0.73]
Dossa, 2015	78321	100216	0.78	[0.78; 0.78]
Farmer, 2016	35529	40722	0.87	[0.87; 0.88]
Graetz, 2020	79750	111463	0.72	[0.71; 0.72]
Guenette, 2015	18	45	0.40	[0.26; 0.56]
Hansen, 2015	1328	1848	0.72	[0.70; 0.74]
Hayashi, 2018	748	754	0.99	[0.98; 1.00]
Hedna, 2013	123	205	0.60	[0.53; 0.67]
Huber, 2016	1854	11035	0.17	[0.16; 0.18]
Iyengar, 2014	82726	150493	0.55	[0.55; 0.55]
Iyengar, 2016	26805	40703	0.66	[0.65; 0.66]
Jarab, 2014	17	37	0.46	[0.29; 0.63]
Jiang, 2020	34242	58349	0.59	[0.58; 0.59]
Koyanagi, 2016	320	452	0.71	[0.66; 0.75]
Leporini, 2016	199	1133	0.18	[0.15; 0.20]
Lopez-Simarro, 2016	158	252	0.63	[0.56; 0.69]
Martinez-Perez, 2021	97	187	0.52	[0.44; 0.59]
Mhatre, 2016	4189	7028	0.60	[0.58; 0.61]
Oh, 2020	36250	37248	0.97	[0.97; 0.97]
Reach, 2018	489	1084	0.45	[0.42; 0.48]
Rinne, 2015	29127	40766	0.71	[0.71; 0.72]
Romagnoli, 2021	9899	29952	0.33	[0.33; 0.34]
Sacks, 2013	32218	49642	0.65	[0.64; 0.65]
Sacks, 2015	6130	12771	0.48	[0.47; 0.49]
Satirapoj, 2020	315	659	0.48	[0.44; 0.52]
Sattler, 2014	84	243	0.35	[0.29; 0.41]
Saundankar, 2016	144216	238402	0.60	[0.60; 0.61]
Schwab, 2019	29733	38614	0.77	[0.77; 0.77]
Sicras-Mainar, 2014	507	987	0.51	[0.48; 0.55]
Simard, 2015	107355	160231	0.67	[0.67; 0.67]
Simard, 2018	34890	52188	0.67	[0.66; 0.67]
Taira, 2017	3819	5163	0.74	[0.73; 0.75]
Tanabe, 2017	4420	7108	0.62	[0.61; 0.63]
Tang, 2020	48922	111186	0.44	[0.44; 0.44]
Tiv, 2012	1158	2859	0.41	[0.39; 0.42]
Vittorino Gaddi, 2014	73828	169375	0.44	[0.43; 0.44]
Walz, 2014	271	430	0.63	[0.58; 0.68]
White, 2012	44	60	0.73	[0.60; 0.84]
Wong, 2012	313759	444418	0.71	[0.70; 0.71]
Wong, 2014	14068	26051	0.54	[0.53; 0.55]
Zhu, 2015	2269	24067	0.09	[0.09; 0.10]
Zongo, 2016	66	153	0.43	[0.35; 0.51]
Total		2424717	0.58	[0.52; 0.65]
Heterogeneity: $\chi^2_{44} = 201623.73$ ($P < .001$), $I^2 = 100\%$				
Total		9798851	0.53	[0.50; 0.57]

Heterogeneity: $\chi^2_{148} = 630992.15$ ($P < .001$), $I^2 = 100\%$

Test for subgroup differences: $\chi^2_{2} = 3.98$ ($P = .14$)

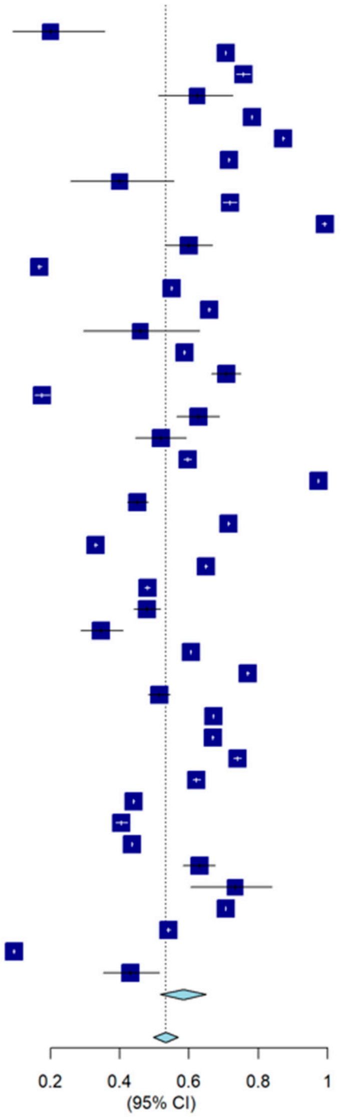

Figure S5. Forest plot of proportions, and relative confidence intervals, of adherent incident patients stratified for follow-up period.

Source	Events	Total	Proportion	95% CI
follow_up = <=12months				
Bell, 2017	15746	27314	0.58	[0.57; 0.58]
Buysman, 2017	1215	2261	0.54	[0.52; 0.56]
Cai, 2016	1471	2925	0.50	[0.48; 0.52]
Cai, 2017	8298	17429	0.48	[0.47; 0.48]
Carls, 2017a	242	652	0.37	[0.33; 0.41]
Carls, 2017b	1878	4985	0.38	[0.36; 0.39]
Chen, 2013	6307	11299	0.56	[0.55; 0.57]
Cheng, 2013	6739	11580	0.58	[0.57; 0.59]
Farmer, 2016	35529	40722	0.87	[0.87; 0.88]
Farr, 2014	46505	134444	0.35	[0.34; 0.35]
Farr, 2016	12362	27385	0.45	[0.45; 0.46]
Gatwood, 2018b	93928	148544	0.63	[0.63; 0.63]
Guenette, 2013	93418	119832	0.78	[0.78; 0.78]
Hong, 2014	10550	23034	0.46	[0.45; 0.46]
Horsburgh, 2019	53592	85066	0.63	[0.63; 0.63]
Kurtyka, 2016	4181	5890	0.71	[0.70; 0.72]
Leporini, 2016	199	1133	0.18	[0.15; 0.20]
Lo-Ciganic, 2015	12589	33130	0.38	[0.37; 0.39]
Lo-Ciganic, 2016	6558	16256	0.40	[0.40; 0.41]
Lokhandwala, 2016	12637	23361	0.54	[0.53; 0.55]
Min, 2019	28005	49424	0.57	[0.56; 0.57]
Nichols, 2016	1890	2844	0.66	[0.65; 0.68]
Nishimura, 2019a	32885	35503	0.93	[0.92; 0.93]
O'Shea, 2013	25540	37238	0.69	[0.68; 0.69]
O'Shea, 2015	7134	11035	0.65	[0.64; 0.66]
Ofori-Asenso, 2019a	10039	15915	0.63	[0.62; 0.64]
Ofori-Asenso, 2019b	7713	11981	0.64	[0.64; 0.65]
Oh, 2020	13668	14284	0.96	[0.95; 0.96]
Quilliam, 2013	54075	73822	0.73	[0.73; 0.74]
Schwab, 2019	29733	38614	0.77	[0.77; 0.77]
Simard, 2015	221119	320462	0.69	[0.69; 0.69]
Tang, 2020	4547	11227	0.41	[0.40; 0.41]
Vichayanrat, 2013	832	1064	0.78	[0.76; 0.81]
Vittorino Gaddi, 2014	9344	22349	0.42	[0.41; 0.42]
Total		1383004	0.59	[0.53; 0.66]

Heterogeneity: χ^2_{33} = 147375.7 (P < .001), I^2 = 100%

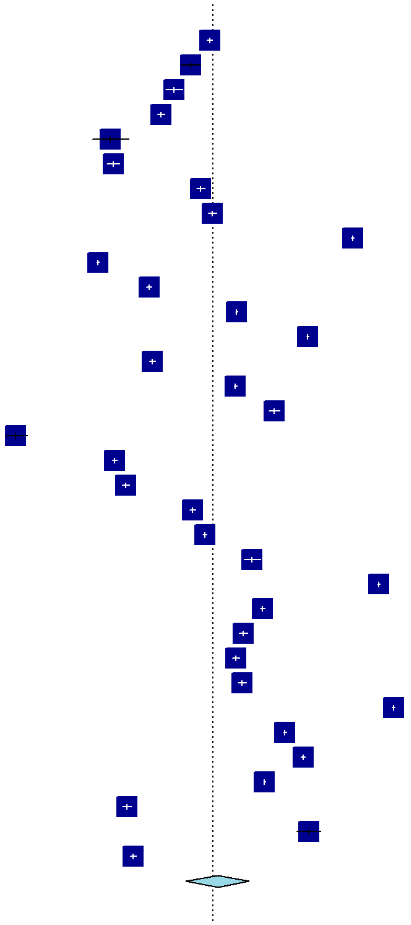

**follow_up = >12months**				
Chen, 2013	47564	79093	0.60	[0.60; 0.60]
Chen, 2016	6398	12123	0.53	[0.52; 0.54]
Cheng, 2013	48071	81060	0.59	[0.59; 0.60]
Farr, 2014	46505	134444	0.35	[0.34; 0.35]
Gatwood, 2018b	87790	148544	0.59	[0.59; 0.59]
Hong, 2014	44248	69102	0.64	[0.64; 0.64]
Horii, 2019	440	884	0.50	[0.46; 0.53]
Horsburgh, 2019	49338	85066	0.58	[0.58; 0.58]
Kim, 2018	22261	65067	0.34	[0.34; 0.35]
Schwab, 2019	22967	31848	0.72	[0.72; 0.73]
Simard, 2015	104150	160231	0.65	[0.65; 0.65]
Total		867462	0.55	[0.48; 0.63]
Heterogeneity: χ^2_{10} = 50460.37 (P < .001), I^2 = 100%				
Total		2250466	0.58	[0.53; 0.64]
Heterogeneity: χ^2_{44} = 213018.13 (P < .001), I^2 = 100%				
Test for subgroup differences: χ^2_1 = 0.61 (P = .43)				

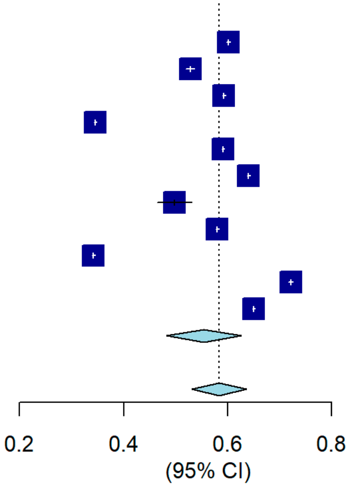

Figure S6. Forest plot of proportions, and relative confidence intervals, of adherent patients stratified for therapeutic class.

Source	Events	Total	Proportion	95% CI
therapeutic_class = biguanides				
Balkhi, 2019	2108	4869	0.43	[0.42; 0.45]
Chepulis, 2020	1207	1595	0.76	[0.73; 0.78]
Chong, 2014	2722	46847	0.06	[0.06; 0.06]
Farmer, 2016	12020	12795	0.94	[0.94; 0.94]
Gatwood, 2018a	64199	105898	0.61	[0.60; 0.61]
Gatwood, 2018b	60678	99148	0.61	[0.61; 0.62]
Guenette, 2016	186	425	0.44	[0.39; 0.49]
Horii, 2019	131	235	0.56	[0.49; 0.62]
Horsburgh, 2019	53592	85066	0.63	[0.63; 0.63]
Huber, 2016	1854	11035	0.17	[0.16; 0.18]
Kim, 2018	5189	14605	0.36	[0.35; 0.36]
Koyanagi, 2016	38	73	0.52	[0.40; 0.64]
Kumar, 2019	554	1000	0.55	[0.52; 0.59]
Min, 2019	28005	49424	0.57	[0.56; 0.57]
Naffaa, 2020	35085	113749	0.31	[0.31; 0.31]
Nichols, 2016	1890	2844	0.66	[0.65; 0.68]
Nishimura, 2019a	7172	7921	0.91	[0.90; 0.91]
O'Shea, 2013	6989	10324	0.68	[0.67; 0.69]
Oung, 2017	174	387	0.45	[0.40; 0.50]
Quilliam, 2013	39842	55043	0.72	[0.72; 0.73]
Ranashinge, 2018	57	134	0.43	[0.34; 0.51]
Romagnoli, 2021	3677	14211	0.26	[0.25; 0.27]
Sacks, 2013	13895	21377	0.65	[0.64; 0.66]
Saraiva, 2020	29	90	0.32	[0.23; 0.43]
Saundankar, 2016	113865	186704	0.61	[0.61; 0.61]
Simard, 2018	34890	52188	0.67	[0.66; 0.67]
Tanabe, 2017	1086	1883	0.58	[0.55; 0.60]
Tang, 2020	48922	111186	0.44	[0.44; 0.44]
White, 2012	24	32	0.75	[0.57; 0.89]
Wong, 2011	5493	6188	0.89	[0.88; 0.90]
Total		1017276	0.55	[0.47; 0.64]

Heterogeneity: χ^2_{29} = 155090.57 (P < .001), I^2 = 100%

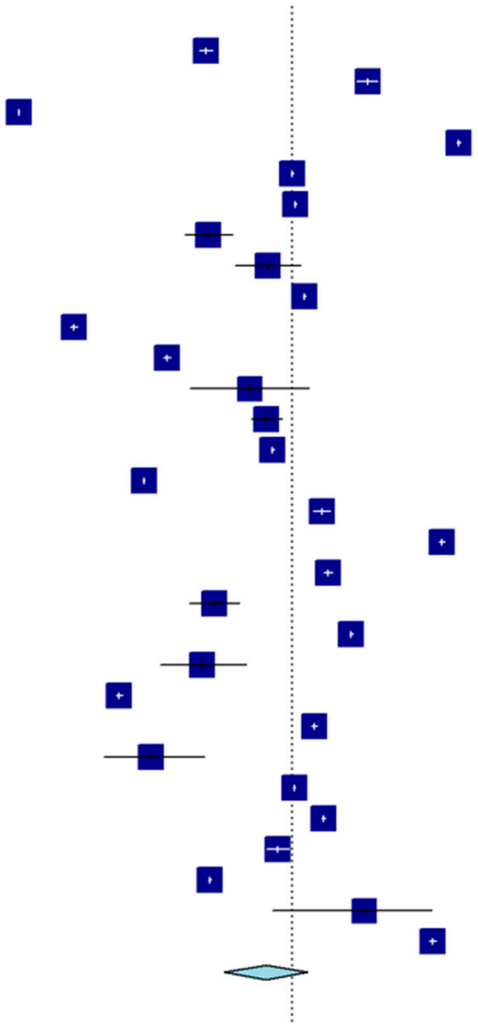

**therapeutic_class = insulin secretagogues**				
Balkhi, 2019	543	947	0.57	[0.54; 0.61]
Bell, 2017	8385	13657	0.61	[0.61; 0.62]
Carls, 2017b	1094	2713	0.40	[0.38; 0.42]
Chong, 2014	13651	26155	0.52	[0.52; 0.53]
Farmer, 2016	9483	10799	0.88	[0.87; 0.88]
Farr, 2014	55597	134961	0.41	[0.41; 0.41]
Gatwood, 2018a	31747	47846	0.66	[0.66; 0.67]
Gatwood, 2018b	29856	44446	0.67	[0.67; 0.68]
Guenette, 2016	6	12	0.50	[0.21; 0.79]
Horii, 2019	120	242	0.50	[0.43; 0.56]
Kim, 2018	9324	28625	0.33	[0.32; 0.33]
Koyanagi, 2016	100	123	0.81	[0.73; 0.88]
Nishimura, 2019a	3859	4108	0.94	[0.93; 0.95]
O'Shea, 2013	1458	2438	0.60	[0.58; 0.62]
Quilliam, 2013	7389	9817	0.75	[0.74; 0.76]
Ranashinge, 2018	36	78	0.46	[0.35; 0.58]
Romagnoli, 2021	2600	6686	0.39	[0.38; 0.40]
Saraiva, 2020	2	22	0.09	[0.01; 0.29]
Saundankar, 2016	88468	131103	0.67	[0.67; 0.68]
Tanabe, 2017	882	1764	0.50	[0.48; 0.52]
Vichayanrat, 2013	832	1064	0.78	[0.76; 0.81]
White, 2012	20	28	0.71	[0.51; 0.87]
Wong, 2011	8680	9777	0.89	[0.88; 0.89]
Total		477411	0.61	[0.52; 0.70]
Heterogeneity: $\chi^2_{22} = 48360.5$ ($P < .001$), $I^2 = 100\%$				
**therapeutic_class = a-glucosidase inhibitors**				
Balkhi, 2019	119	205	0.58	[0.51; 0.65]
Horii, 2019	99	208	0.48	[0.41; 0.55]
Koyanagi, 2016	25	65	0.38	[0.27; 0.51]
Nishimura, 2019a	3372	3763	0.90	[0.89; 0.91]
Romagnoli, 2021	444	1723	0.26	[0.24; 0.28]
Saundankar, 2016	492	1100	0.45	[0.42; 0.48]
Tanabe, 2017	655	1081	0.61	[0.58; 0.64]
Total		8145	0.53	[0.36; 0.69]
Heterogeneity: $\chi^2_{6} = 2766.8$ ($P < .001$), $I^2 = 100\%$				

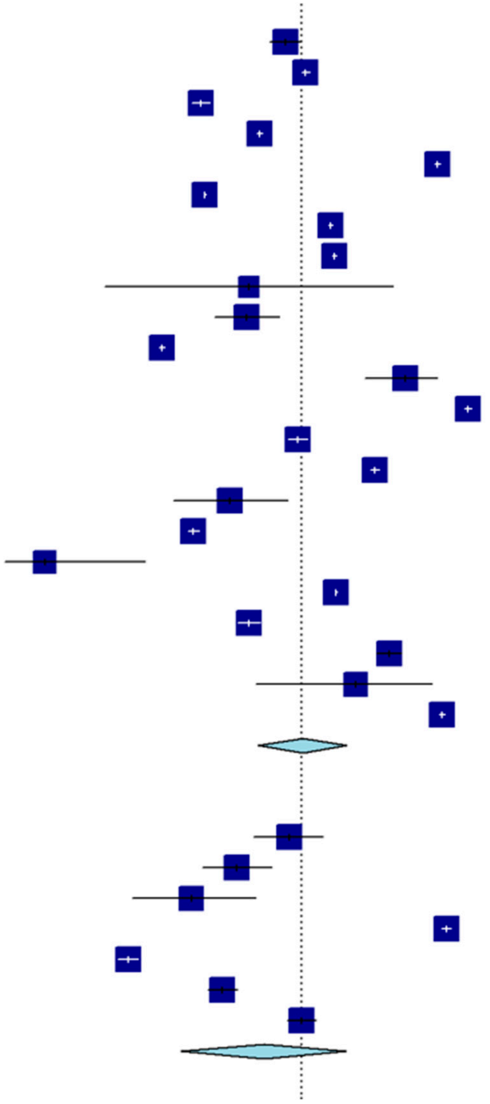

**therapeutic_class = TZDs**				
Balkhi, 2019	430	662	0.65	[0.61; 0.69]
Chong, 2014	1383	2553	0.54	[0.52; 0.56]
Farmer, 2016	8906	9765	0.91	[0.91; 0.92]
Farr, 2014	15425	42012	0.37	[0.36; 0.37]
Gatwood, 2018a	2329	3061	0.76	[0.75; 0.78]
Gatwood, 2018b	2204	2863	0.77	[0.75; 0.79]
Horii, 2019	55	130	0.42	[0.34; 0.51]
Nishimura, 2019a	1117	1244	0.90	[0.88; 0.91]
Quilliam, 2013	6844	8962	0.76	[0.75; 0.77]
Romagnoli, 2021	460	637	0.72	[0.69; 0.76]
Sacks, 2013	4366	7212	0.61	[0.59; 0.62]
Saundankar, 2016	30253	43608	0.69	[0.69; 0.70]
Tanabe, 2017	309	592	0.52	[0.48; 0.56]
Total		123301	0.68	[0.58; 0.77]
Heterogeneity: $\chi^2_{12} = 19315.86$ ($P < .001$), $I^2 = 100\%$				
**therapeutic_class = SGLT-2i**				
Bell, 2017	7361	13657	0.54	[0.53; 0.55]
Buysman, 2017	1215	2261	0.54	[0.52; 0.56]
Cai, 2016	1471	2925	0.50	[0.48; 0.52]
Cai, 2017	4969	9633	0.52	[0.51; 0.53]
Nishimura, 2019a	965	1168	0.83	[0.80; 0.85]
Ofori-Asenso, 2019b	7713	11981	0.64	[0.64; 0.65]
Romagnoli, 2021	674	839	0.80	[0.77; 0.83]
Singhal, 2019	356	750	0.47	[0.44; 0.51]
Total		43214	0.61	[0.51; 0.71]
Heterogeneity: $\chi^2_{7} = 1126.69$ ($P < .001$), $I^2 = 99\%$				

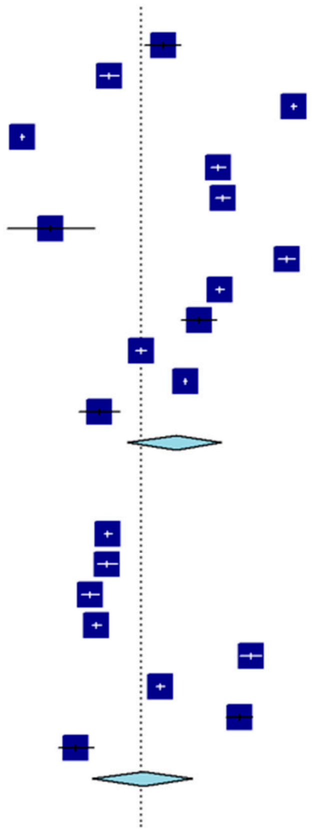

therapeutic_class = DPP-4i				
Balkhi, 2019	770	1285	0.60	[0.57; 0.63]
Cai, 2017	3329	7796	0.43	[0.42; 0.44]
Carls, 2017a	242	652	0.37	[0.33; 0.41]
Carls, 2017b	784	2272	0.35	[0.33; 0.37]
Farmer, 2016	4871	5363	0.91	[0.90; 0.92]
Farr, 2014	29070	61399	0.47	[0.47; 0.48]
Farr, 2016	12362	27385	0.45	[0.45; 0.46]
Horii, 2019	165	301	0.55	[0.49; 0.61]
Koyanagi, 2016	119	148	0.80	[0.73; 0.86]
Kurtyka, 2016	4181	5890	0.71	[0.70; 0.72]
Nigam, 2012	4241	7306	0.58	[0.57; 0.59]
Nishimura, 2019a	30535	32155	0.95	[0.95; 0.95]
Ofori-Asenso, 2019a	10039	15915	0.63	[0.62; 0.64]
Oh, 2020	36250	37248	0.97	[0.97; 0.97]
Rascati, 2017	13040	26089	0.50	[0.49; 0.51]
Romagnoli, 2021	1373	1754	0.78	[0.76; 0.80]
Sacks, 2013	1064	1812	0.59	[0.56; 0.61]
Saundankar, 2016	5547	7681	0.72	[0.71; 0.73]
Tanabe, 2017	1488	1788	0.83	[0.81; 0.85]
Total		244239	0.66	[0.56; 0.75]
Heterogeneity: χ^2_{18} = 73084.72 (P < .001), I^2 = 100%				
Total		1913586	0.61	[0.56; 0.65]
Heterogeneity: χ^2_{99} = 320865.57 (P < .001), I^2 = 100%				
Test for subgroup differences: χ^2_5 = 5.40 (P = .37)				

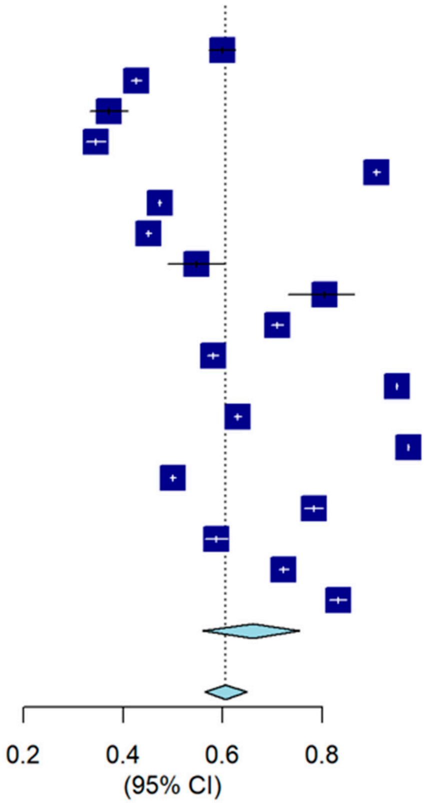

Figure S7. Forest plot of proportions, and relative confidence intervals, of adherent patients stratified for therapeutic regimen complexity.

Source	Events	Total	Proportion	95% CI
therapy = Monotherapy				
Abdullah, 2019	63	112	0.56	[0.47; 0.66]
Ahmad, 2013	70	136	0.51	[0.43; 0.60]
Aminde, 2019	43	80	0.54	[0.42; 0.65]
An, 2014	125	159	0.79	[0.71; 0.85]
Balkhi, 2019	3970	7968	0.50	[0.49; 0.51]
Cai, 2017	8298	17429	0.48	[0.47; 0.48]
Chepulis, 2020	579	782	0.74	[0.71; 0.77]
Chew, 2015b	107	197	0.54	[0.47; 0.61]
Gordon, 2018	19524	23925	0.82	[0.81; 0.82]
Heissam, 2015	51	134	0.38	[0.30; 0.47]
Horsburgh, 2019	53592	85066	0.63	[0.63; 0.63]
Horvat, 2018	93	169	0.55	[0.47; 0.63]
Huber, 2016b	1854	11035	0.17	[0.16; 0.18]
Jamous, 2011	11	33	0.33	[0.18; 0.52]
Koyanagi, 2016	42	90	0.47	[0.36; 0.57]
Kumar, 2019	554	1000	0.55	[0.52; 0.59]
Kurtyka, 2016	1931	2874	0.67	[0.65; 0.69]
Nishimura, 2019	46959	50349	0.93	[0.93; 0.93]
O'Shea, 2013	7971	12762	0.62	[0.62; 0.63]
O'Shea, 2015	7134	11035	0.65	[0.64; 0.66]
Quilliam, 2013	54075	73822	0.73	[0.73; 0.74]
Romagnoli, 2021	9228	37361	0.25	[0.24; 0.25]
Saraiva, 2020	31	112	0.28	[0.20; 0.37]
Shields, 2020	19664	24400	0.81	[0.80; 0.81]
Tunceli, 2015	78764	133499	0.59	[0.59; 0.59]
Tunceli, 2015b	3772	6276	0.60	[0.59; 0.61]
Vichayanrat, 2013	832	1064	0.78	[0.76; 0.81]
Vittorino Gaddi, 2014	40329	91816	0.44	[0.44; 0.44]
Waari, 2018	58	110	0.53	[0.43; 0.62]
Total		593795	0.57	[0.50; 0.64]

Heterogeneity: $\chi^2_{28} = 93386.88$ ($P < .001$), $I^2 = 100\%$

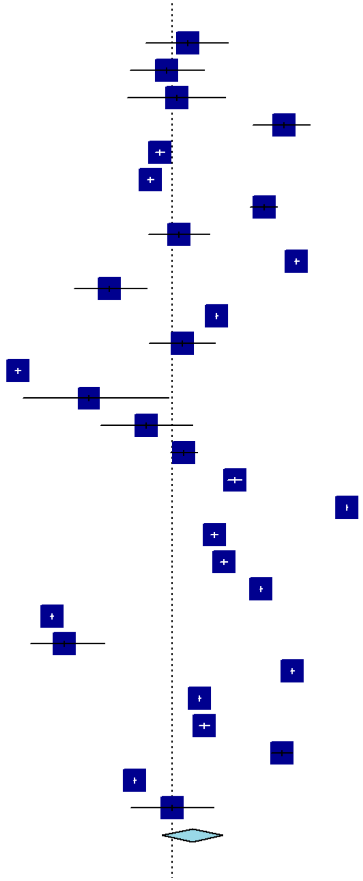

therapy = Combination

Abdullah, 2019	65	120	0.54	[0.45; 0.63]
Ahmad, 2013	158	337	0.47	[0.41; 0.52]
Aminde, 2019	26	54	0.48	[0.34; 0.62]
An, 2014	347	449	0.77	[0.73; 0.81]
Balkhi, 2019	180	410	0.44	[0.39; 0.49]
Basak, 2014	3006	6043	0.50	[0.48; 0.51]
Bloomgarden, 2017	10674	18560	0.58	[0.57; 0.58]
Chepulis, 2020	379	501	0.76	[0.72; 0.79]
Chew, 2015	224	385	0.58	[0.53; 0.63]
Gordon, 2018	8023	9924	0.81	[0.80; 0.82]
Heissam, 2015	22	217	0.10	[0.06; 0.15]
Horvat, 2018	76	154	0.49	[0.41; 0.58]
Jamous, 2011	39	97	0.40	[0.30; 0.51]
Koyanagi, 2016	82	144	0.57	[0.48; 0.65]
Kurtyka, 2016	2250	3016	0.75	[0.73; 0.76]
Lokhandwala, 2016	12637	23361	0.54	[0.53; 0.55]
Min, 2019	28005	49424	0.57	[0.56; 0.57]
Nishimura, 2019b	6691	7524	0.89	[0.88; 0.90]
O'Shea, 2013	2012	3196	0.63	[0.61; 0.65]
Romagnoli, 2021	671	4102	0.16	[0.15; 0.18]
Saraiva, 2020	23	138	0.17	[0.11; 0.24]
Sicras-Mainar, 2014	507	987	0.51	[0.48; 0.55]
Vittorino Gaddi, 2014	33499	51247	0.65	[0.65; 0.66]
Vlacho, 2021	36118	75809	0.48	[0.47; 0.48]
Waari, 2018	74	180	0.41	[0.34; 0.49]
Zhu, 2015	683	7454	0.09	[0.09; 0.10]
Total		263833	0.51	[0.42; 0.60]

Heterogeneity: $\chi^2_{25} = 24347.42$ ($P < .001$), $I^2 = 100\%$

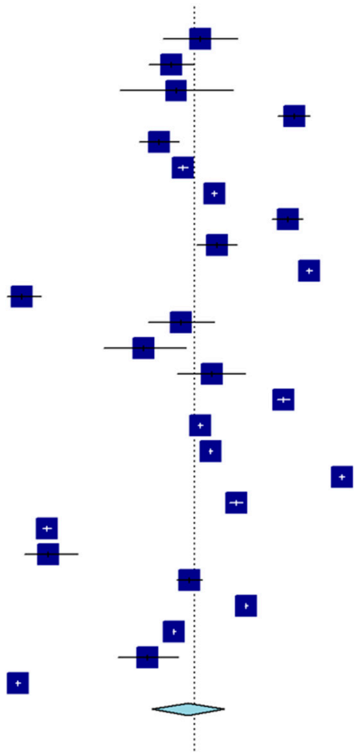

**therapy = OADs + Insulin**				
Abdullah, 2019	46	82	0.56	[0.45; 0.67]
Acharya, 2019	15	39	0.38	[0.23; 0.55]
Adisa, 2013	11	25	0.44	[0.24; 0.65]
Afaya, 2020	42	50	0.84	[0.71; 0.93]
Ahmad, 2013	34	84	0.40	[0.30; 0.52]
Aminde, 2019	11	32	0.34	[0.19; 0.53]
An, 2014	84	116	0.72	[0.63; 0.80]
Arulmozhi, 2014	13	34	0.38	[0.22; 0.56]
Ashur, 2015	208	324	0.64	[0.59; 0.69]
Bonger, 2018	23	24	0.96	[0.79; 1.00]
Chepulis, 2020	249	312	0.80	[0.75; 0.84]
Elsous, 2017	25	43	0.58	[0.42; 0.73]
Gatwood, 2018a	16577	24421	0.68	[0.67; 0.68]
Horvat, 2018	25	35	0.71	[0.54; 0.85]
Jarab, 2014	30	116	0.26	[0.18; 0.35]
Lee, 2013	14	39	0.36	[0.21; 0.53]
Leporini, 2016	44	151	0.29	[0.22; 0.37]
Lo-Ciganic, 2016	252	862	0.29	[0.26; 0.32]
Mirahmadizadeh, 2020	24	142	0.17	[0.11; 0.24]
Mukherjee, 2013	75	173	0.43	[0.36; 0.51]
Saraiva, 2020	14	50	0.28	[0.16; 0.42]
Tandon, 2015	0	7	0.00	[0.00; 0.41]
Waari, 2018	65	161	0.40	[0.33; 0.48]
Total		27322	0.48	[0.38; 0.58]
Heterogeneity: $\chi^2_{22} = 1111.56$ ($P < .001$), $I^2 = 98\%$				
Total		884950	0.53	[0.48; 0.58]
Heterogeneity: $\chi^2_{77} = 121850.17$ ($P < .001$), $I^2 = 100\%$				
Test for subgroup differences: $\chi^2_{2} = 2.53$ ($P = .28$)				

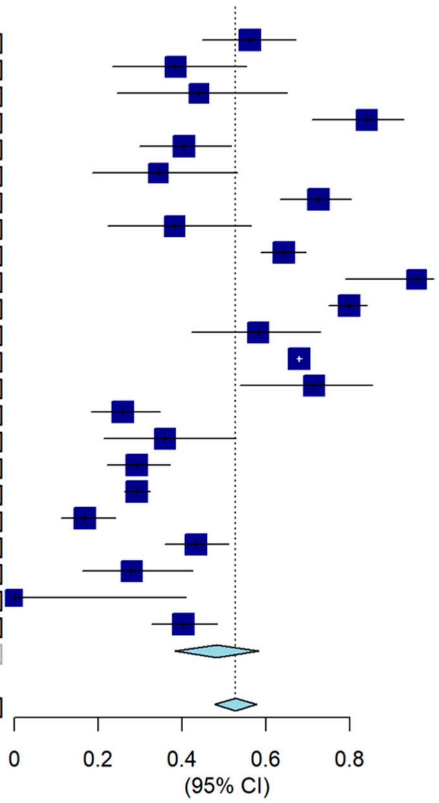

Figure S8. Forest plot of proportions, and relative confidence intervals, of adherent patients stratified for method of adherence measurement.

Source	Events	Total	Proportion	95% CI
data_collection_cat = Questionnaires and verbal interviews				
Abdullah, 2019	67	117	0.57	[0.48; 0.66]
Acharya, 2019	50	161	0.31	[0.24; 0.39]
Adisa, 2013	60	130	0.46	[0.37; 0.55]
Aditama, 2019	8	40	0.20	[0.09; 0.36]
Afaya, 2020	209	247	0.85	[0.80; 0.89]
Ahmad, 2013	245	473	0.52	[0.47; 0.56]
Aloudah, 2018	158	395	0.40	[0.35; 0.45]
AlQarni, 2019	76	212	0.36	[0.29; 0.43]
Aminde, 2019	69	134	0.51	[0.43; 0.60]
Andanalusia, 2019	5	60	0.08	[0.03; 0.18]
Arulmozhi, 2014	61	116	0.53	[0.43; 0.62]
Ashur, 2015	126	199	0.63	[0.56; 0.70]
Ayoub, 2019	196	500	0.39	[0.35; 0.44]
Benrazavy, 2019	245	417	0.59	[0.54; 0.64]
Bonger, 2018	141	151	0.93	[0.88; 0.97]
Bruce, 2015	77	200	0.38	[0.32; 0.46]
Chew, 2015	349	605	0.58	[0.54; 0.62]
de Vries, 2014	53	85	0.62	[0.51; 0.73]
Degefa, 2020	116	116	1.00	[0.97; 1.00]
Elsous, 2017	136	241	0.56	[0.50; 0.63]
Fai, 2017	23	115	0.20	[0.13; 0.28]
Guenette, 2015	18	45	0.40	[0.26; 0.56]
Guenette, 2016	261	598	0.44	[0.40; 0.48]
Haines, 2018	77	126	0.61	[0.52; 0.70]
Hayashi, 2018	748	754	0.99	[0.98; 1.00]
Heissam, 2015	98	372	0.26	[0.22; 0.31]
Ibrahim, 2021	167	304	0.55	[0.49; 0.61]
Jamous, 2011	50	131	0.38	[0.30; 0.47]
Jannuzzi, 2014	12	17	0.71	[0.44; 0.90]
Jannuzzi, 2020	115	157	0.73	[0.66; 0.80]
Jarab, 2014	17	37	0.46	[0.29; 0.63]
Khan, 2014	18	184	0.10	[0.06; 0.15]
Kim, 2016	78	304	0.26	[0.21; 0.31]
Kumar, 2019	554	1000	0.55	[0.52; 0.59]
Lee, 2013	84	278	0.30	[0.25; 0.36]
Lee, 2017	164	382	0.43	[0.38; 0.48]
Leporini, 2016	199	1133	0.18	[0.15; 0.20]
Lopez-Simarro, 2016	158	252	0.63	[0.56; 0.69]
Mallah, 2019	101	300	0.34	[0.28; 0.39]
Martinez-Perez, 2021	97	187	0.52	[0.44; 0.59]
Mirahmadizadeh, 2020	44	358	0.12	[0.09; 0.16]
Mroueh, 2018	78	245	0.32	[0.26; 0.38]
Mukherjee, 2013	156	225	0.69	[0.63; 0.75]
Olickal, 2021	299	515	0.58	[0.54; 0.62]
Pascal, 2012	87	120	0.72	[0.64; 0.80]
Peeters, 2015	15	21	0.71	[0.48; 0.89]
Rahmatullah, 2021	66	198	0.33	[0.27; 0.40]
Rana, 2017	54	145	0.37	[0.29; 0.46]
Ranashinge, 2018	93	212	0.44	[0.37; 0.51]
Rathish, 2019	46	200	0.23	[0.17; 0.29]
Reach, 2011	81	90	0.90	[0.82; 0.95]
Reach, 2018	489	1084	0.45	[0.42; 0.48]
Sankar, 2015	75	317	0.24	[0.19; 0.29]
Saraiva, 2020	54	250	0.22	[0.17; 0.27]
Satirapoj, 2020	315	659	0.48	[0.44; 0.52]
Shrestha, 2013	54	90	0.60	[0.49; 0.70]
Tandon, 2015	17	66	0.26	[0.16; 0.38]
Tiv, 2012	1158	2859	0.41	[0.39; 0.42]
Vichayanrat, 2013	832	1064	0.78	[0.76; 0.81]
Vietri, 2016	779	2030	0.38	[0.36; 0.41]
Waari, 2018	67	129	0.52	[0.43; 0.61]
Walz, 2014	271	430	0.63	[0.58; 0.68]
Wong, 2015	383	565	0.68	[0.64; 0.72]
Wu, 2016	59	130	0.45	[0.37; 0.54]
Zhou, 2013	39	87	0.45	[0.34; 0.56]
Zongo, 2016	66	153	0.43	[0.35; 0.51]
Total		23517	0.49	[0.43; 0.55]

Heterogeneity: $\chi^2_{65} = 4784.23$ ($P < .001$), $I^2 = 99\%$

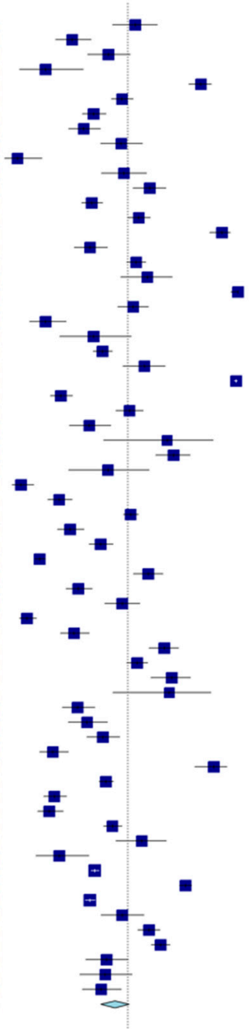

data_collection_cat = Administrative data and pill counting methods

An, 2014	388	492	0.79	[0.75; 0.82]
Balkhi, 2019	2652	5457	0.49	[0.47; 0.50]
Basak, 2014	3006	6043	0.50	[0.48; 0.51]
Bell, 2017	15746	27314	0.58	[0.57; 0.58]
Bloomgarden, 2017	10674	18560	0.58	[0.57; 0.58]
Bryson, 2013	313759	444418	0.71	[0.70; 0.71]
Buysman, 2017	1215	2261	0.54	[0.52; 0.56]
Cai, 2016	1471	2925	0.50	[0.48; 0.52]
Cai, 2017	8298	17429	0.48	[0.47; 0.48]
Carls, 2017a	242	652	0.37	[0.33; 0.41]
Carls, 2017b	1878	4985	0.38	[0.36; 0.39]
Chang, 2015	184035	559864	0.33	[0.33; 0.33]
Chen, 2013	6307	11299	0.56	[0.55; 0.57]
Chen, 2016	6398	12123	0.53	[0.52; 0.54]
Cheng, 2013	6739	11580	0.58	[0.57; 0.59]
Chepulis, 2020	1207	1595	0.76	[0.73; 0.78]
Chong, 2014	42756	75555	0.57	[0.56; 0.57]
Dossa, 2015	78321	100216	0.78	[0.78; 0.78]
Farmer, 2016	35529	40722	0.87	[0.87; 0.88]
Farr, 2014	100092	238372	0.42	[0.42; 0.42]
Farr, 2016	12362	27385	0.45	[0.45; 0.46]
Garcia, 2019	13	279	0.05	[0.03; 0.08]
Gatwood, 2018a	99430	159032	0.63	[0.62; 0.63]
Gatwood, 2018b	93928	148544	0.63	[0.63; 0.63]
Gatwood, 2021	58	433	0.13	[0.10; 0.17]
Gordon, 2018	27547	33849	0.81	[0.81; 0.82]
Graetz, 2020	79750	111463	0.72	[0.71; 0.72]
Guenette, 2013	93418	119832	0.78	[0.78; 0.78]
Hagen, 2014	2820	4978	0.57	[0.55; 0.58]
Hansen, 2015	1328	1848	0.72	[0.70; 0.74]
Hedna, 2013	123	205	0.60	[0.53; 0.67]
Hong, 2014	10550	23034	0.46	[0.45; 0.46]
Horii, 2019	440	884	0.50	[0.46; 0.53]
Horsburgh, 2019	53592	85066	0.63	[0.63; 0.63]
Horvat, 2018	128	272	0.47	[0.41; 0.53]
Huber, 2016	1854	11035	0.17	[0.16; 0.18]
Iyengar, 2014	82726	150493	0.55	[0.55; 0.55]
Iyengar, 2016	26805	40703	0.66	[0.65; 0.66]
Jiang, 2020	34242	58349	0.59	[0.58; 0.59]
Kharjul, 2019	3246	7138	0.45	[0.44; 0.47]
Kim, 2018	22261	65067	0.34	[0.34; 0.35]
Koyanagi, 2016	320	452	0.71	[0.66; 0.75]
Kurtyka, 2016	4181	5890	0.71	[0.70; 0.72]
Lo-Ciganic, 2015	12589	33130	0.38	[0.37; 0.39]
Lo-Ciganic, 2016	6558	16256	0.40	[0.40; 0.41]
Lokhandwala, 2016	12637	23361	0.54	[0.53; 0.55]
Manteuffel, 2014	3391734	5141047	0.66	[0.66; 0.66]
Mhatre, 2016	4189	7028	0.60	[0.58; 0.61]
Miller, 2015	695	1998	0.35	[0.33; 0.37]
Min, 2019	28005	49424	0.57	[0.56; 0.57]
Naffaa, 2020	35085	113749	0.31	[0.31; 0.31]
Nichols, 2016	1890	2844	0.66	[0.65; 0.68]
Nichols, 2018	45218	54480	0.83	[0.83; 0.83]
Nigam, 2012	4241	7306	0.58	[0.57; 0.59]
Nishimura, 2019a	46959	50349	0.93	[0.93; 0.93]
Nishimura, 2019b	6691	7524	0.89	[0.88; 0.90]
Ofori-Asenso, 2019a	10039	15915	0.63	[0.62; 0.64]
Ofori-Asenso, 2019b	7713	11981	0.64	[0.64; 0.65]
Oh, 2020	36250	37248	0.97	[0.97; 0.97]
O'Shea, 2013	10644	15958	0.67	[0.66; 0.67]
O'Shea, 2015	7134	11035	0.65	[0.64; 0.66]
Oung, 2017	174	387	0.45	[0.40; 0.50]
Quilliam, 2013	54075	73822	0.73	[0.73; 0.74]
Rascati, 2017	13040	26089	0.50	[0.49; 0.51]
Rinne, 2015	29127	40766	0.71	[0.71; 0.72]
Romagnoli, 2021	9899	29952	0.33	[0.33; 0.34]
Sacks, 2013	32218	49642	0.65	[0.64; 0.65]
Sacks, 2015	6130	12771	0.48	[0.47; 0.49]
Sattler, 2014	84	243	0.35	[0.29; 0.41]
Saundankar, 2016	144216	238402	0.60	[0.60; 0.61]
Schwab, 2019	29733	38614	0.77	[0.77; 0.77]
Shields, 2020	19664	24400	0.81	[0.80; 0.81]
Sicras-Mainar, 2014	507	987	0.51	[0.48; 0.55]
Simard, 2015	107355	160231	0.67	[0.67; 0.67]
Simard, 2018	34890	52188	0.67	[0.66; 0.67]
Simon-Tuval, 2018	158	308	0.51	[0.46; 0.57]
Singhal, 2019	356	750	0.47	[0.44; 0.51]
Taira, 2017	3819	5163	0.74	[0.73; 0.75]
Tanabe, 2017	4420	7108	0.62	[0.61; 0.63]
Tang, 2020	48922	111186	0.44	[0.44; 0.44]
Tunceli, 2015a	78764	133499	0.59	[0.59; 0.59]
Tunceli, 2015b	3772	6276	0.60	[0.59; 0.61]
Vittorino Gaddi, 2014	73828	169375	0.44	[0.43; 0.44]
Vlacho, 2021	36118	75808	0.48	[0.47; 0.48]
White, 2012	44	60	0.73	[0.60; 0.84]
Wong, 2011	23997	26782	0.90	[0.89; 0.90]
Wong, 2012	313759	444418	0.71	[0.70; 0.71]
Wong, 2014	14068	26051	0.54	[0.53; 0.55]
Zhu, 2015	2269	24067	0.09	[0.09; 0.10]
Zomahoun, 2016	293	340	0.86	[0.82; 0.90]
Total		10018411	0.58	[0.54; 0.62]
Heterogeneity: χ^2_{89} = 667229.15 (P < .001), I^2 = 100%				
Total		10041928	0.54	[0.51; 0.58]
Heterogeneity: χ^2_{155} = 673989.71 (P < .001), I^2 = 100%				
Test for subgroup differences: χ^2_1 = 6.42 (P = .01)				

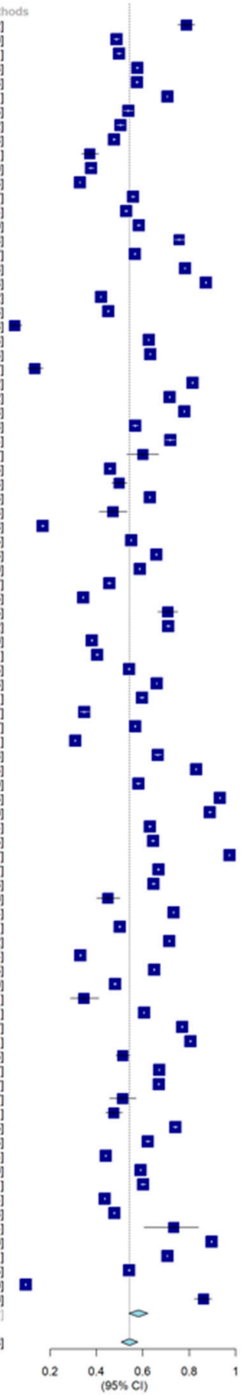

Supplement: Supplementary file 1 [file jcm-12-01981-s001.zip › jcm-2229937-supplementary.pdf]
